# Dengue infection in India: A systematic review and meta-analysis

**DOI:** 10.1371/journal.pntd.0006618

**Published:** 2018-07-16

**Authors:** Parasuraman Ganeshkumar, Manoj V. Murhekar, Veeraraghavadoss Poornima, Velusamy Saravanakumar, Krishnendu Sukumaran, Anandan Anandaselvasankar, Denny John, Sanjay M. Mehendale

**Affiliations:** 1 Department of Epidemiology, National Institute of Epidemiology, Chennai, Tamil Nadu, India; 2 School of Public Health, Jawaharlal Institute of Postgraduate Medical Education and Research, Puducherry, India; 3 Campbell Collaboration, New Delhi, India; 4 Division of Epidemiology and Communicable Diseases, Indian Council of Medical Research, New Delhi, India; University of California San Francisco, UNITED STATES

## Abstract

**Introduction:**

Dengue is the most extensively spread mosquito-borne disease; endemic in more than 100 countries. Information about dengue disease burden, its prevalence, incidence and geographic distribution is critical in planning appropriate control measures against dengue fever. We conducted a systematic review and meta-analysis of dengue fever in India

**Methods:**

We searched for studies published until 2017 reporting the incidence, the prevalence or case fatality of dengue in India. Our primary outcomes were (a) prevalence of laboratory confirmed dengue infection among clinically suspected patients, (b) seroprevalence in the general population and (c) case fatality ratio among laboratory confirmed dengue patients. We used binomial–normal mixed effects regression model to estimate the pooled proportion of dengue infections. Forest plots were used to display pooled estimates. The metafor package of R software was used to conduct meta-analysis.

**Results:**

Of the 2285 identified articles on dengue, we included 233 in the analysis wherein 180 reported prevalence of laboratory confirmed dengue infection, seven reported seroprevalence as evidenced by IgG or neutralizing antibodies against dengue and 77 reported case fatality. The overall estimate of the prevalence of laboratory confirmed dengue infection among clinically suspected patients was 38.3% (95% CI: 34.8%–41.8%). The pooled estimate of dengue seroprevalence in the general population and CFR among laboratory confirmed patients was 56.9% (95% CI: 37.5–74.4) and 2.6% (95% CI: 2–3.4) respectively. There was significant heterogeneity in reported outcomes (p-values<0.001).

**Conclusions:**

Identified gaps in the understanding of dengue epidemiology in India emphasize the need to initiate community-based cohort studies representing different geographic regions to generate reliable estimates of age-specific incidence of dengue and studies to generate dengue seroprevalence data in the country.

## Author summary

Dengue fever, an extensively spread mosquito-borne disease, is endemic in more than 100 countries. Information about dengue disease burden, its prevalence and incidence and geographic distribution is necessary to guide in planning appropriate control measures including the dengue vaccine that has recently been licensed in a few countries. We performed a systematic review and meta-analysis of published studies in India on dengue. The overall estimate of the prevalence of laboratory confirmed dengue infection based on testing of more than 200,000 clinically suspected patients from 180 Indian studies was 38.3%. The pooled estimate of dengue seroprevalence in the general population and CFR among laboratory confirmed dengue patients was 56.9% and 2.6% respectively. There were no community-based studies reporting incidence of dengue. Our review also identified certain knowledge gaps about dengue epidemiology in the country. Identified gaps in the understanding of dengue epidemiology in India emphasize the need to initiate community-based cohort studies representing different geographic regions to generate reliable estimates of age-specific incidence of dengue and studies to generate dengue seroprevalence data in the country.

## Introduction

Dengue is the most extensively spread mosquito-borne disease, transmitted by infected mosquitoes of Aedes species. Dengue infection in humans results from four dengue virus serotypes (DEN-1, DEN-2, DEN-3, and DEN-4) of *Flavivirus* genus. As per the WHO 1997 classification, symptomatic dengue virus infection has been classified into dengue fever (DF), dengue haemorrhagic fever (DHF) and dengue shock syndrome (DSS). The revised WHO classification of 2009 categorizes dengue patients according to different levels of severity as dengue without warning signs, dengue with warning signs (abdominal pain, persistent vomiting, fluid accumulation, mucosal bleeding, lethargy, liver enlargement, increasing haematocrit with decreasing platelets) and severe dengue [1,2,3]. Dengue fever is endemic in more than 100 countries with most cases reported from the Americas, South-East Asia and Western Pacific regions of WHO [1]. In India, dengue is endemic in almost all states and is the leading cause of hospitalization. Dengue fever had a predominant urban distribution a few decades earlier, but is now also reported from peri-urban as well as rural areas [4,5]. Surveillance for dengue fever in India is conducted through a network of more than 600 sentinel hospitals under the National Vector Borne Disease Control Program (NVBDCP) [6], Integrated Disease Surveillance Program (IDSP) [7] and a network of 52 Virus Research and Diagnostic Laboratories (VRDL) established by Department of Health Research [8]. In 2010, an estimated 33 million cases had occurred in the country [9]. During 2016, the NVBDCP reported more than 100,000 laboratory confirmed cases of dengue [6]. It is therefore possible that dengue disease burden is grossly under-estimated in India.

High dengue disease burden and frequent outbreaks result in a serious drain on country’s economy and stress on the health systems. In India, case detection, case management, and vector control are the main strategies for prevention and control of dengue virus transmission [6]. A new dengue vaccine is now available and several vaccines are in the process of development [10, 11, 12]. Information about dengue disease burden, its prevalence, incidence and geographic distribution is necessary in decisions on appropriate utilization of existing and emerging prevention and control strategies. With this background, we conducted a systematic review and meta-analysis to estimate the disease burden of dengue fever in India. We also reviewed serotype distribution of dengue viruses in circulation, and estimated case fatality ratios as well as proportion of secondary infections.

## Methods

### Search strategy and selection criteria

This systematic review is registered in PROSPERO (Reg. No. CRD 42017065625). We searched Medline (PubMed), Cochrane Central, WHOLIS, Scopus, Science Direct, Ovid, Google Scholar, POPLINE, Cost-Effectiveness Analysis (CEA) Registry and Paediatric Economic Database Evaluation (PEDE) databases for articles published up to 2017. The main search terms included incidence, prevalence, number of reported cases, mortality, disease burden, cost of illness, or economic burden of dengue in India. The complete search strategy is described in S1 Appendix. Back referencing of included studies in bibliography was also done to identify additional studies.

### Review approach

The search results were initially imported to Zotero software (Version 4.0.29.5) and duplicate records were removed. During title screening, we examined relevant studies from various databases. Our inclusion criterion was studies reporting dengue infection in India, not restricted to setting, design, purpose and population. Titles thus selected were subjected to abstract screening. Studies were considered eligible for further examination in full text if their abstracts reported incidence, prevalence, number of reported cases, mortality or the burden of dengue fever anywhere in India. Studies reporting complications of dengue, serotype details of dengue virus as well as seroprevalence of dengue were also included. Using a pre-designed data extraction form, two reviewers extracted details from selected studies independently. The data, which differed between the reviewers, were resolved by consensus. Information about the year of publication, study setting (hospital/laboratory based, or community-based), study location, study period, laboratory investigations, number of suspected patients tested and positives, age distribution of cases, and details of dengue serotypes were abstracted (S1 Dataset).

The primary outcome measures of interest were (a) prevalence (proportion) of laboratory confirmed dengue infection among clinically suspected patients in hospital/laboratory based or community-based studies, (b) seroprevalence of dengue in the general population and (c) case fatality ratio among laboratory confirmed dengue patients. The diagnosis of acute dengue infection among the clinically suspected patients was based on any of the following laboratory criteria: (a) detection of non-structural protein-1 (NS1) antigen, (b) Immunoglobulin M (IgM) antibodies against dengue virus (c) haemagglutination inhibition (HI) antibodies against dengue virus, (d) Real-time polymerase chain reaction (RT-PCR) positivity or (e) virus isolation. Seroprevalence of dengue was based on detection of IgG or neutralizing antibodies against dengue virus. Studies providing prevalence (proportion) of laboratory confirmed dengue infection among clinically suspected patients were classified into (a) hospital/laboratory-based surveillance studies and (b) outbreak investigations or hospital/laboratory-based surveillance studies when the outbreak was ongoing in the area, as mentioned in the original research paper. Studies regarding outbreak investigations considered an increase in number of reported cases of febrile illness in a geographical area, as the criteria for defining an outbreak. The outbreak investigations included one or more of the following activities: active search for case-patients in the community, calculation of attack rates for suspected case-patients, confirmation of aetiology and entomological investigations. For the case fatality ratio, the numerator included reported number of deaths due to dengue and denominator as laboratory confirmed dengue patients.

Our secondary outcomes of interest were the following: (a) proportion of primary and secondary infections among the laboratory confirmed dengue patients. This classification was made based on the information about dengue serology provided in the paper. Primary dengue infection was defined as acute infection, as indicated by qualitative detection of NS1 antigen, and/or IgM or HI antibodies or RT-PCR positivity and absence of IgG antibodies against dengue virus. A case of acute infection as defined above, in presence of IgG antibodies, was considered as secondary dengue infection [2,13,14]. Some of the studies used the ratio of IgG to IgM antibodies as the criteria for differentiating primary and secondary infections [14]; (b) distribution of predominant and co-circulating dengue virus serotypes; (c) proportion of severe dengue infections based on WHO 1997 or WHO 2009 criteria [1,2]. The category of severe dengue infection included patients with DHF and DSS as per the WHO 1997 classification as well as severe dengue infections classified as per the WHO 2009 classification and (d) cost of illness, which included reported direct and indirect costs associated with dengue hospitalization.

### Risk of bias

The risk of bias was assessed using a modified Joanna Briggs Institute (JBI) appraisal checklist for studies reporting prevalence data [15] and essential items listed in the Strengthening the Reporting of Observational Studies in Epidemiology (STROBE) checklist [16]. The criteria for assessing bias primarily included methods for selecting participants, methods for laboratory testing, and outcome variables (Supplementary file S2 Appendix).

### Statistical analysis

We conducted quantitative synthesis to derive meta-estimates of primary and secondary outcomes (severity of disease and primary/ secondary infections) and qualitative synthesis to describe the serotype distribution and economic burden due to dengue. We followed Meta-analysis of Observational Studies in Epidemiology (MOOSE) guidelines [17]. For each study, primary outcomes (prevalence of acute infection, seroprevalence and CFR) were summarized as proportion and their 95% confidence intervals were computed. We used logit and inverse logit transformations for variance stabilization of proportions [18]. Binomial–Normal mixed effects regression model was used to estimate the pooled proportion of dengue infections. Forest plots were used to display pooled estimates. Heterogeneity was tested using likelihood ratio test. Funnel plots with logit prevalence on x-axis and standard errors on y-axis and Egger’s test were used to evaluate publication bias. Independent variables potentially associated with the prevalence of laboratory confirmed dengue were included as fixed-effects in univariate and multivariate binomial meta-regression models. P <0.05 was considered statistically significant. Sensitivity analysis was carried out by leaving out one study at a time in the order of publication to check for consistency of pooled estimates. Analyses were performed in the R statistical programming language using the ‘metafor’ package [19,20].

## Results

### Characteristics of included studies

**Fig 1:**
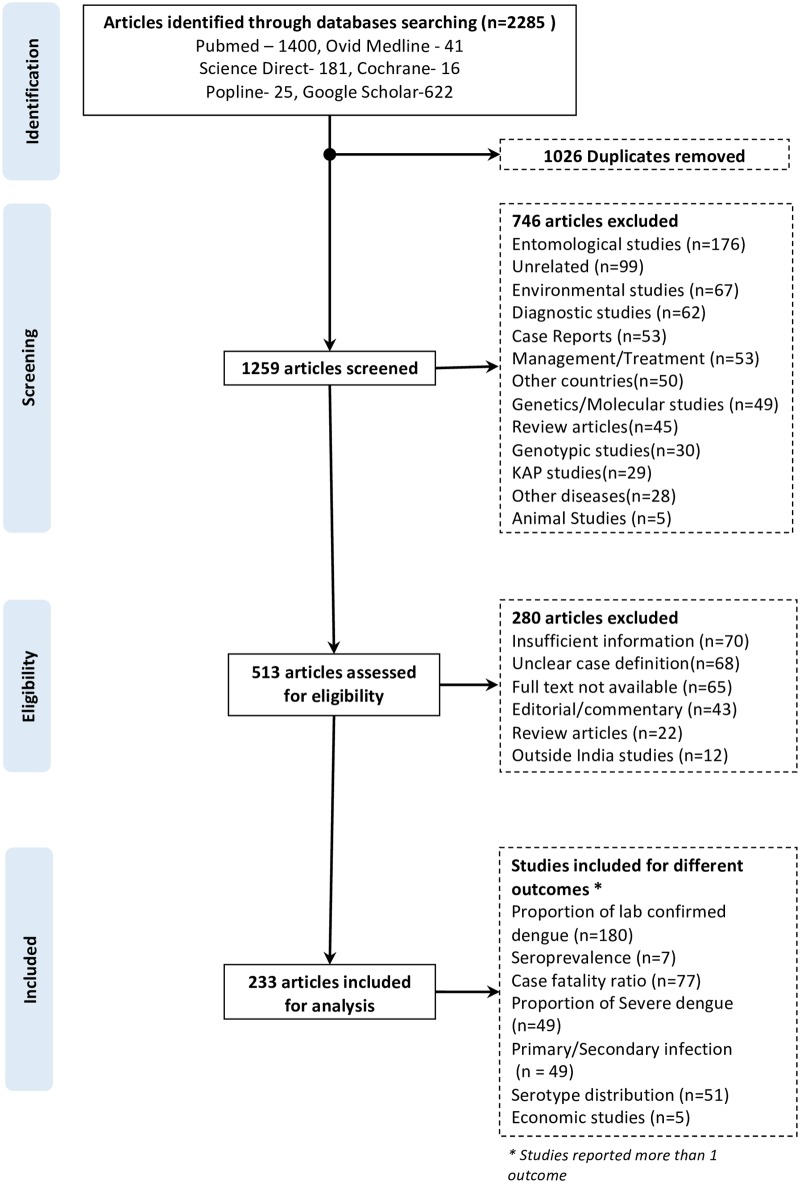
Flow diagram showing the article selection in the systematic review on dengue in India.

The search strategy initially identified 2,285 articles from different databases. After removal of duplicates, 1,259 articles were considered for title and abstract screening. Seven hundred and forty-six articles were excluded for reasons provided in Fig 1. Thus, 513 articles were found to be eligible for full-text review. After the review of full-text articles, 233 studies were included for the analysis [21–253]. The details of the studies included in the review are provided in the PRISMA flowchart (Fig 1). None of the studies reported incidence of dengue fever.

### Primary outcomes

#### Prevalence (proportion) of laboratory confirmed dengue fever

Of the 233 studies included in the analysis, 180 provided information about proportion of laboratory confirmed dengue cases among clinically suspected patients [21–200]. This included 154 studies conducted in hospital or laboratory setting [21–174] and 26 studies reporting outbreak investigations [175–200]. Of the 154 studies conducted in hospital/ laboratory setting, 40 were conducted when an outbreak was ongoing in the area [135–74]. The diagnosis of acute dengue infection was based on a single assay in 86 studies (IgM antibodies = 68, RT-PCR = 11, HI antibodies = 4, virus isolation = 2, detection of NS1 antigen = 1) and more than one assay in 95 studies.

*Case definitions used*: Of the 154 studies conducted in hospital settings, WHO or NVBDCP case definitions were used by 39 and 2 studies respectively. The remaining studies used case definitions such as acute febrile illness/acute undifferentiated illness (n = 20), and clinically suspected dengue fever (n = 93). Similarly, of the 26 reported outbreaks, investigators used WHO or NVBDCP case definitions in 7 and 2 settings respectively, whereas acute febrile illness and clinically suspected dengue fever case definitions were used in 5 and 12 settings respectively.

*Place and time distribution of studies*: Of the 154 studies conducted in hospital setting, 75, 41, 27 and 7 were from north, south, east and western Indian states respectively, whereas 3 studies were from north-eastern states. One study reported data from VRDL network, covering multiple regions in India [65]. Of the 26 outbreaks, most (10, 38.5%) were reported from Southern states, followed by 9 (34.6%) in the north, 4 (15.4%) in the east, and 3 (11.5%) in the north-eastern Indian states. Most (65, 42.2%) studies conducted in hospital settings were between 2011–2017, while 48 (31.2%) were conducted between 2006–2010 and 41 (26.6%) were conducted before 2006. Eighteen (69.2%) of the 26 outbreaks were reported after 2000.

Of the 180 studies which reported proportion of dengue cases, 74 studies (30%) provided the details of laboratory confirmed cases by month with most (n = 60, 81%) reporting higher dengue positivity between August and November months.

**Fig 2:**
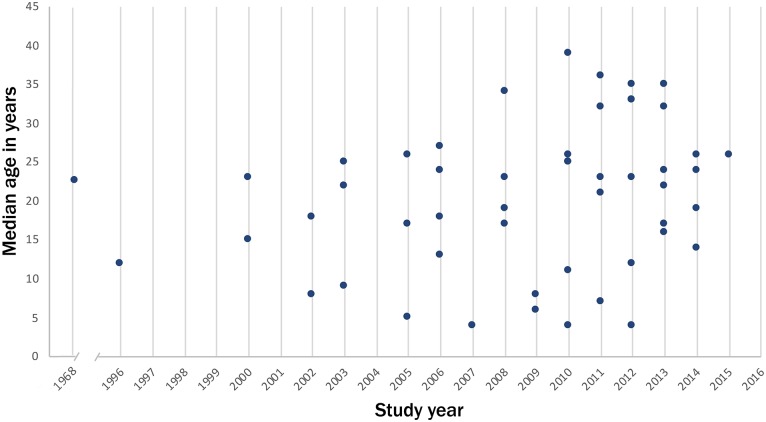
Distribution of median age of laboratory confirmed dengue cases by year of study, India.

*Age distribution of dengue cases*: The age distribution of laboratory confirmed dengue patients was available from 52 out of 180 studies. The pooled median age of laboratory confirmed dengue cases in these studies was 22 years (Fig 2). Fifteen (28.8%) studies reported the median age of dengue cases below 15 years.

**Fig 3:**
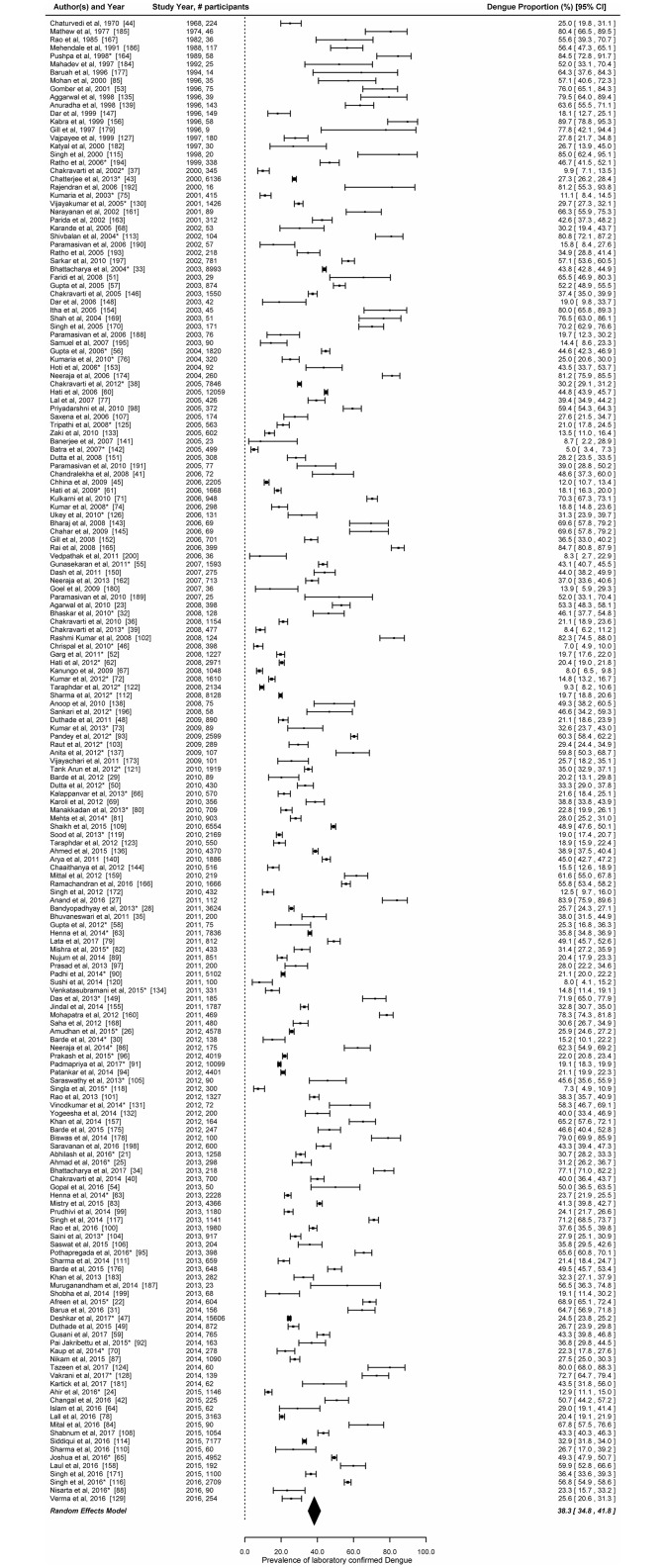
Prevalence (proportion) of laboratory confirmed dengue among clinically suspected patients in India. Error bars indicate 95% confidence intervals. Diamonds show the pooled estimates with 95% confidence intervals based on random effects (RE) model.

*Estimates of prevalence (proportion)*: The overall estimate of the prevalence of laboratory confirmed dengue infection in the random effects model based on testing of 213,285 clinically suspected patients from 180 studies was 38.3% (95% CI: 34.8%–41.8%) (Fig 3). There was a significant heterogeneity in the prevalence reported by the 180 studies (LRT p<0.001). The prevalence of laboratory confirmed dengue infection was higher in studies reporting outbreaks or hospital-based surveillance studies during outbreaks (47.3%, 95% CI: 40.9–53.8) as compared to hospital-based surveillance studies (33.6%, 95% CI: 29.9–37.5) (S1A and S1B Fig). The attack rates of suspected dengue case patients were available in 8 out of the 26 outbreak investigations reports. The attack rates ranged between 1.9% and 19.5%.

In the univariate mixed effect meta-regression model, odds of laboratory confirmation were higher in case of outbreaks or hospital-based studies conducted during outbreaks (OR = 1.8, 95% CI: 1.3–2.4). Studies which used WHO/ NVBDCP case definitions for enrolment of patients also had higher odds of detecting laboratory confirmed dengue compared to studies which used acute febrile illness/ clinically suspected dengue cases as case definitions. Compared to studies conducted before 2006–10, studies conducted between 2011 and 2017 had higher odds of identifying laboratory confirmed patients (OR = 1.33, 95% CI: 0.93–1.9). The odds of laboratory confirmation did not differ by region (Table 1). In the multivariate meta-regression model constructed by including all covariates, case definition (WHO/NVBDCP), type of study (hospital-based surveillance studies conducted during outbreaks or outbreaks) and period of study (prior to 2005 and 2011–2017) were associated with higher odds of dengue cases being laboratory confirmed.

**Table 1:** Meta-regression of variables associated with the proportion of laboratory confirmed dengue infections, based on a univariate and multivariate model. (n = 180).

Variable	Univariate analysis		Multivariate analysis	
	Odds Ratio (95% CI)	P*	Odds Ratio (95% CI)	P*
**Region**				
East	Ref		Ref	
North	1.41 (0.93,2.15)	0.11	1.30 (0.89,1.98)	0.17
North-East	2.39 (0.97,5.88)	0.06	1.94 (0.81,4.65)	0.14
South	1.34 (0.85,2.11)	0.20	1.23 (0.79,1.90)	0.36
West	0.82 (0.36,1.88)	0.65	0.93 (0.42,2.06)	0.85
**Study Type**				
Hospital based surveillance (HBS)	Ref		Ref	
Outbreak/HBS during outbreak	1.78 (1.32,2.41)	0.00	1.65 (1.20,2.27)	0.00
**Case Definition**				
AFI /Clinically suspected	Ref		Ref	
WHO/NVBDCP	1.52 (1.09,2.12)	0.01	1.38 (1.01,1.91)	0.05
**Year of study (midpoint)**	0.97 (0.95,0.99)			
**Year of study (midpoint)**				
1982–2005	1.67 (1.14–2.47)	0.00	1.46 (1.01–2.14)	0.05
2006–2010	Ref		Ref	
2011–2017	1.33 (0.93–1.9)	0.12	1.45 (1.02–2.05)	0.04

Ref—Reference category; CI—Confidence interval; P*–P value.

#### Seroprevalence of dengue among healthy individuals

**Fig 4:**
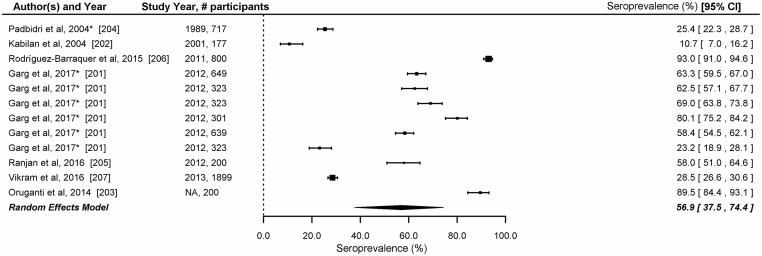
Seroprevalence of dengue in India. Error bars indicate 95% confidence intervals. Diamonds show the pooled estimates with 95% confidence intervals based on random effects (RE) model.

We included 7 studies reporting seroprevalence of dengue based on detection of IgG (n = 5), neutralizing antibodies (n = 1) or HI antibodies (n = 1) against dengue in the analysis [201–207]. These studies, conducted in 12 Indian states [Andaman and Nicobar islands (n = 1), Andhra Pradesh (n = 2), Tamil Nadu (n = 3), Delhi (n = 4), West Bengal (n = 1), and Maharashtra (n = 1)], surveyed 6,551 individuals. The study population surveyed in these studies included healthy children (n = 2), general population (n = 3), blood donors (n = 1) and neighbourhood contacts of dengue confirmed cases (n = 1). The overall seroprevalence of dengue fever based on these studies was 56.9% (95% CI: 37.5–74.4) (Fig 4). The age-specific prevalence of IgG antibodies was available in three studies [201, 204, 206]. There was a significant heterogeneity in the seroprevalence reported by the seven studies (LRT p<0.001). In the 3 studies which provided age specific seroprevalence, by the age of 9 years, 47.6% -73.4% children were reported to have developed IgG or neutralizing antibodies against dengue (Table 2).

**Table 2:** Dengue seroprevalence by age, reported in 3 studies from India.

Garg et al (n = 2558) [201]		Rodríguez-Barraquer et al (n = 800) [206]		Padbidri et al (n = 717) [204]	
Age (y)	Seroprevalence (%)	Age (y)	Seroprevalence (%)	Age (y)	Sero-prevalence (%)
5	40.7%	5–9	77.1	0–9	47.6
6	50.9%	10–14	90.3	10–19	24.0
7	58.6%	15–19	91.7	20–29	26.8
8	67.4%	20–29	96.3	30–39	25.0
9	70.8%	30–40	98.8	> = 40	23.3
10	73.4%				
**Overall**	**59.6 (95% CI: 57·7–61·5)**		**93 (95% CI: 91.1–94.6)**		**25.4 (95%CI: 22.3–28.7)**

Figure in square bracket indicate reference

#### Case fatality ratios (CFR)

**Fig 5:**
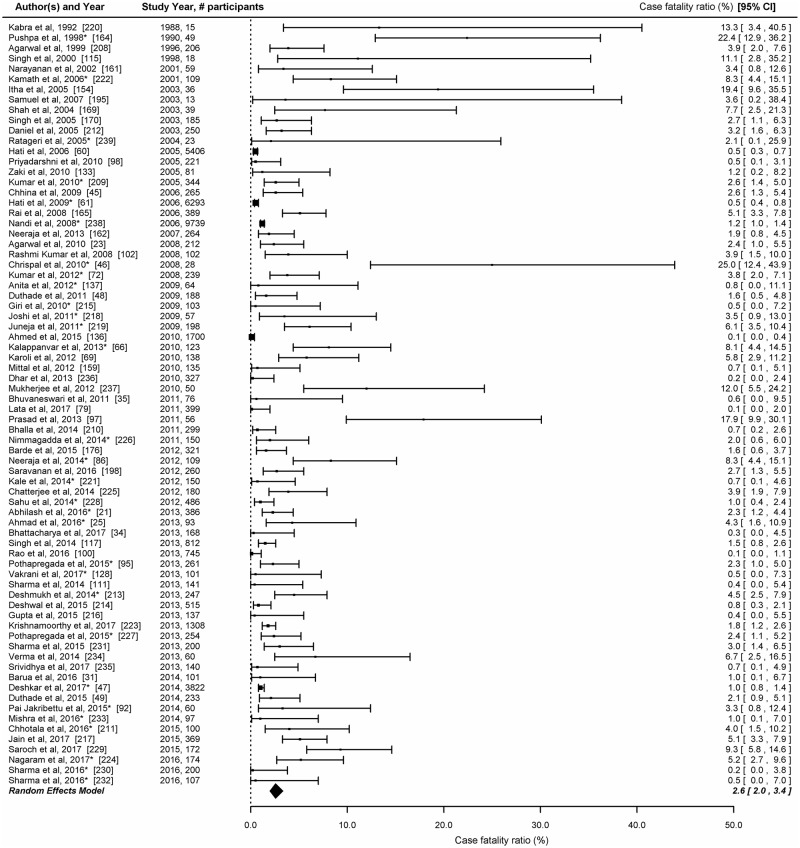
Studies reporting case fatality ratio among laboratory confirmed dengue cases in India. Error bars indicate 95% confidence intervals. Diamonds show the pooled estimates with 95% confidence intervals based on random effects (RE) model.

Seventy-seven studies provided information about case fatality ratios; most of them (n = 72, 93.5%) were conducted after 2000. The reported CFRs in these studies ranged from 0% to 25%. There was a significant heterogeneity in the CFRs reported by the 74 studies (LRT p<0.001). Twenty (25.9%) studies reported CFR of 2% or more. Three studies [30, 239, 195] which affected overall meta-estimates due to small denominator and hence were excluded from analysis. The pooled estimate of CFR was 2.6% (95% CI: 2.0–3.4) (Fig 5).

### Secondary outcomes

#### Primary and secondary dengue infection

**Fig 6:**
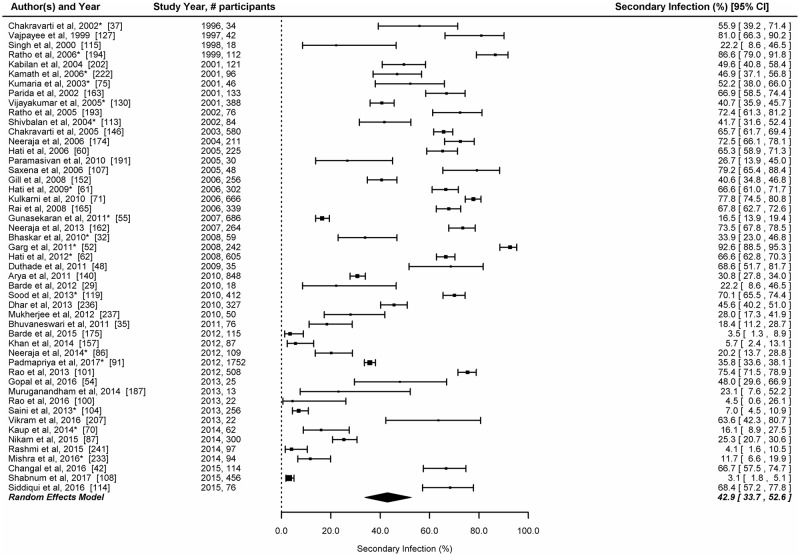
Proportion of secondary infection among laboratory confirmed dengue cases in India. Error bars indicate 95% confidence intervals. Diamonds show the pooled estimates with 95% confidence intervals based on random effects (RE) model.

A total of 49 studies provided data which enabled classification of laboratory confirmed dengue into primary and secondary dengue infections. The number of patients with acute dengue infections in these studies ranged between 13 and 1752. Only two studies estimated the proportion of secondary infection based on IgG to IgM ratio [174, 237]. The prevalence of secondary dengue infection was <10% in 6 studies, 10–25% in 9 studies, 26–50% in 12 studies, 51–75% in 17 studies and >75% in 5 studies. The overall proportion of secondary dengue infection among laboratory confirmed patients was 42.9% (95%CI: 33.7–52.6) (Fig 6).

#### Proportion of severe cases

**Fig 7:**
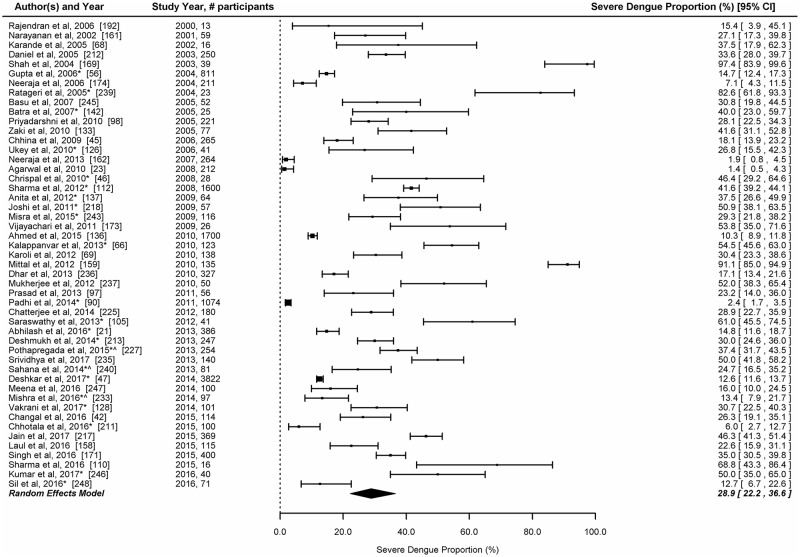
Proportion of severe dengue infections among laboratory confirmed dengue cases in India.

Information about severity of dengue was available in 49 studies. Most studies (n = 46, 93.9%) used the WHO 1997 classification while 3 studies used the WHO 2009 classification for dengue severity. The reported proportion of severe dengue cases among laboratory confirmed patients ranged between 1.4% and 97.4%. The overall proportion of severe dengue among laboratory confirmed studies in the random effects model was 28.9% (95% CI: 22.2–36.6) (Fig 7).

#### Serotypes of dengue virus

Information about dengue serotypes was available in 51 studies. These studies were conducted in 19 Indian states; with a regional distribution of north (n = 28), south (n = 13), east (n = 4), northeast (n = 4), and west (n = 2). Thirty-eight (75%) of the 51 studies reported circulation of more than one serotype. The predominant serotypes reported in these studies were DEN-2 and DEN-1 in the northern region, DEN-2 and DEN-3 in the southern region, and DEN-1 and DEN-2 in the eastern and the western regions. In the four studies reported from the north-eastern region, the predominant serotypes was DEN-3 followed by DEN-1 and DEN-2 serotypes (Table 3).

**Table 3:** Circulating dengue virus serotypes by state and region, India, 1982–2015.

	2000 and earlier				2001 to 2005					2006 to 2010					2011 and above				
State by region	1982	1988	1996	1997	2001	2002	2003	2004	2005	2006	2007	2008	2009	2010	2011	2012	2013	2014	2015
**East**																			
Odisha [149]														2,3					
Odisha [90]														2,1					
West Bengal [123]														1,2					
West Bengal [67]												1,4,3,2							
**North**																			
Chandigarh [193]						2													
Delhi [167]	1,2																		
Delhi [147]			2,1																
Delhi [127]				1															
Delhi [148]							3,1,2,4												
Delhi [107]									2,3										
Delhi[143]										3,1,2,4									
Delhi [36]												1,2,3							
Delhi [172]														1					
Delhi [42]																			1,2,4
Delhi [64]																			2,1,3,4
Delhi [217]																			2,4
Delhi [22]															2,1,3			2,1,3	
Delhi [76]						2,3,4,1													
Delhi [38]						3,2,1,4													
Delhi [56]							3,1,2,4												
Delhi [39]											1,2,3,4								
Delhi [242]											1,2,3,4								
Delhi [137]												2,4							
Delhi [58]														1					
Madhya Pradesh [163]					2														
Madhya Pradesh [29]														4					
Madhya Pradesh [176]																	2		
Madhya Pradesh [30]															4,1				
Uttar Pradesh [243]							2,3,1,4												
Uttar Pradesh [93]												2,3,1							
Uttar Pradesh [82]													2,3,1						
Uttar Pradesh [96]															1,3,2				
**North-east**																			
Arunachal Pradesh [157]																3, 1,2			
Assam, Nagaland, Meghalaya, Manipur [50]													1,2,3,4						
Manipur [183]																	3,1,2,4		
Manipur [196]											2								
**South**																			
Andaman & Nicobar [181]																		3	
Andaman & Nicobar [144]														1,2					
Andhra Pradesh [150]											4, 3								
Karnataka [131]															2,3,4,1				
Kerala [138]												2							
Kerala [244]																	1,3,2		
Kerala [73]												2,3,1,4							
Kerala [80]												1,3,2							
Puducherry [27]															3				
Puducherry [153]							3												
Tamil Nadu [189]											3								
Telangana [162]											4,3								
Telangana [86]															2,3,4,1				
**West**																			
Maharashtra [186]		2,1																	
Maharashtra [98]									1,2,3										

Key for coloured cell: Blue—One circulating serotype, Yellow—two co-circulating serotypes, Green—three co- circulating serotypes, Orange—four co- circulating serotypes. Numbers mentioned in the cell indicate predominant serotypes, in descending order.

#### Economic burden

*Direct and Indirect cost analysis*: An estimate of direct and indirect costs was reported in three studies. The average direct cost per case of dengue ranged between USD 23.5 and USD 161 and the indirect cost was around USD 25 whereas the average cost of hospitalization ranged between USD 186 and USD 432.2 [range 249-252]. The cost of dengue treatment in the private health sector was two to four times higher than that in the public sector hospitals [249, 253].

*Economic impact of dengue on National Economy*: Three macro-level studies addressed the economic impact of dengue faced by India [250, 251, 253]. It was estimated that the average total economic burden due to dengue in India was USD 27.4 million [251]. Another study estimated that the total direct medical cost of dengue in 2012 was USD 548 million [253]. The overall economic burden of dengue would be even higher if the cost borne by individual patients is combined with the society level cost of dengue prevention, vector control, disease control and its management, dengue surveillance as well as the cost of research and development [250, 251, 253].

### Publication bias and sensitivity analysis

Funnel plots and Egger’s test revealed no publication bias in the estimates of dengue prevalence in hospital-based surveillance studies, hospital-based surveillance studies during outbreaks and outbreak investigations. CFR estimates, however, showed a significant publication bias, and studies with high prevalence were more likely to be published. In the sensitivity analysis, the estimated pooled proportions were found to be consistent for all study outcomes. (S3 Appendix)

## Discussion

The present study has estimated the burden of dengue fever based on published literature from India spanning over five decades. Most of the published literature included in the analysis were hospital/ laboratory-based surveillance studies or reports of dengue outbreak investigations. Additionally the published data from VRDL network has been included in the analysis [65, 96]. The data from the other two nationally representative surveillance platforms could not be used for the analysis because surveillance data from NVBDCP only reports the number of laboratory confirmed dengue cases, while the IDSP data is not available in the public domain.

There was no community-based epidemiological study reporting the incidence of dengue fever. Our analysis revealed that among the clinically suspected dengue fever patients, the estimated prevalence of laboratory-confirmed dengue infection was 38%. The burden of dengue was also variable in studies conducted in different settings. Our findings indicated that most of the laboratory confirmed dengue cases in India occurred in young adults. Dengue positivity was higher between the months of August and November, corresponding to monsoon and post-monsoon season in most states in India.

In the meta-regression, studies that had used WHO/NVBDCP case definitions and the hospital based studies conducted during outbreaks or studies reporting outbreaks were more likely to have laboratory confirmation of dengue. The odds of laboratory confirmation were also higher among studies conducted during the period of 2011 to 2017, as compared to studies conducted prior to the year 2000.

Information about seroprevalence of dengue in the general population is a useful indicator for measuring endemicity of dengue fever. The dengue vaccine (CYD-TDV) manufactured by Sanofi Pasteur has been introduced in two sub-national programs in Philippines and Brazil [254] and it has been suggested that vaccine acts by boosting the naturally acquired immunity [255]. WHO SAGE conditionally recommends the use of this vaccine for areas in which dengue is highly endemic as defined by seroprevalence in the population targeted for vaccination [12, 256]. The results of the two vaccine trials and mathematical modelling suggest that optimal benefits of vaccination if seroprevalence in the age group targeted for vaccination was in the range of ≥70% [255, 256]. In 2018, WHO revised the recommendation from population sero-prevalence criteria to pre-vaccination screening strategy [257]. The pooled estimate based on the seven studies conducted in India indicated a dengue seroprevalence of 57%. However, this estimated seroprevalence is not representative of the country, as these studies were conducted only in 12 Indian states, and some had used a convenience sampling method [201].

The computed pooled estimate of case fatality due to dengue in India was 2.6% with a high variability in the reported CFRs. The CFR estimated in our study was higher than the estimate of 1.14% (95% CI: 0.82–1.58) reported in the meta-analysis of 77 studies conducted globally; in the 69 studies which adopted WHO 1997 dengue case classification, the pooled CFR was 1.1% (0.8–1.6) while the pooled CFR for 8 studies which used the WHO 2009 case definition, the pooled CFR was 1.6% (95% CI: 0.64–4.0) [258]. Higher CFR observed in our analysis could be due to smaller sample sizes as 14 of the 35 studies that reported CFR of 2.6 or higher had a sample size of 100 or less, while in the remaining 21 studies the denominator ranging between 101 and 400. Also, we only considered laboratory confirmed dengue cases in the denominator for the calculation of CFR. As per the NVBDCP surveillance data, a total of 683,545 dengue cases and 2,576 deaths were reported in India during 2009–2017 giving a CFR of 0.38% [6]. The lower CFR estimates from NVBDCP data could probably be on account of under-reporting of deaths due to dengue, or inclusion of higher number of mild cases in the denominator [259]. As per the NVBDCP surveillance data, an average of 28,227 dengue cases and 154 deaths were reported annually during 2009–2012. The number of dengue cases reported increased thereafter, with an average of 100,690 cases per year during 2013–2017. However, the reported number of deaths did not increase proportionately. The information about severity of dengue cases is not available from NVBDCP surveillance data.

The published studies from India indicated circulation of all the four-dengue serotypes, with DEN-2 and DEN-3 being the more commonly reported serotypes. Two third of the studies reported circulation of more than one serotype. Co-circulation of multiple serotypes was particularly evident from the published studies in Delhi. More than two third (16/19) studies from Delhi reported circulation of more than one serotype; and most of the studies conducted in the last 10 years identified co-circulation of more than one serotype [Table 3]. Our review also revealed that more than two-fifth of the laboratory confirmed infections were secondary dengue infections and nearly one-fourth of the cases were severe in nature. Circulation of numerous dengue serotypes is known to increase the probability of secondary infection, leading to a higher risk of severe dengue disease [260].

Our systematic review has certain limitations. First, our study included only peer-reviewed literature from selected databases and we excluded grey literature which may have provided additional data. Second, most of the studies on disease burden were hospital-based, with no community-based studies estimating incidence. Hospital-based studies do not provide any information about the community level transmission as hospitalization is a function of health-seeking behaviour of the population. In absence of the information about health seeking behaviour provided in these studies, we estimated the prevalence of dengue using number of patients tested in the hospitals as the denominator. Third, the hospital-based studies used varying case definitions and laboratory tests to confirm dengue infection. Fourth, information about the type of health facility (public or private), or residential status of patients (urban or rural), and age was not uniformly reported and hence we did not estimate the dengue prevalence by these variables.

In conclusion, the findings of our systematic review indicate that dengue continues to be an important public health problem in India, as evidenced by the high proportion of dengue positivity, severity and case fatality as well as co-circulation of multiple dengue virus serotypes. Our review also identified certain research gaps in the understanding on dengue epidemiology in the country. There is a need to initiate well planned community-based cohort studies representing different geographic regions of the country in order to generate reliable estimates of age-specific incidence of dengue fever in India. As such studies are cost intensive, a national level survey to estimate age-stratified dengue seroprevalence rates could be an alternative. Such estimates could be used to derive the relative proportions of primary and secondary infections using mathematical models [261]. Well planned studies in different geographic settings are also needed to generate reliable data about economic burden from India. Although the existing dengue surveillance platforms of NVBDCP, IDSP and VRDL are generating data about dengue disease burden, these systems could be strengthened to also generate data about dengue serotypes, severity, and primary and secondary infection from India.

## Supporting information

S1 Appendix Search criteria. (PDF)

S2 Appendix Critical appraisal checklist for quality assessment of studies. (PDF)

S3 Appendix Publication bias and sensitivity analysis. (PDF)

S1 Dataset Abstracted data of included studies. (XLSX)

S1 Fig Prevalence (proportion) of laboratory confirmed dengue among clinically suspected patients by study type. (PDF)

S1 Checklist PRISMA checklist. (DOC)
