# Supplementary material for: Dengue infection in India: A systematic review and meta-analysis
Source: PLoS Negl Trop Dis. 2018 Jul 16;12(7):e0006618. doi: 10.1371/journal.pntd.0006618 (PMC6078327; doi:10.1371/journal.pntd.0006618)
Supplement: S1 Appendix — (PDF) [file pntd.0006618.s001.pdf]

Search strategy

Search for	Search string
Incidence	((Incidence[Title/Abstract]) AND (((((((((((((((((((((((((((((((((((India [Title/Abstract] OR Kerala[tiab] OR Tamil Nadu [tiab] OR Andaman and Nicobar[tiab] OR Arunachal Pradesh [tiab] OR Assam [tiab] OR Bihar[tiab] OR Chandigarh [tiab] OR Chattisgarh[tiab] OR Dadra and Nagar Haveli[tiab] OR Daman and Diu[tiab] OR Delhi [tiab] OR Goa [tiab] OR Gujarat [tiab] OR Haryana [tiab] OR Himachal Pradesh [tiab] OR Jammu and Kashmir [tiab] OR Jharkhand [tiab] OR Karnataka[tiab] OR Lakshadweep[tiab] OR Madhya Pradesh[tiab] OR Maharashtra[tiab] OR Manipur[tiab] ORMeghalaya[tiab] OR Mizoram [tiab]OR Nagaland[tiab] OR Odisha[tiab] OR Puducherry [tiab] OR Punjab[tiab] OR Rajasthan[tiab] OR Sikkim [tiab] OR Telangana[tiab] OR Tripura [tiab] OR Uttar Pradesh [tiab] OR West Bengal[tiab]))))))))))))))))))))))))))))))))))) AND Dengue[MeSH Major Topic]
Prevalence	((Prevalence[Title/Abstract]) AND (((((((((((((((((((((((((((((((((((India [Title/Abstract] OR Kerala[tiab] OR Tamil Nadu [tiab] OR Andaman and Nicobar[tiab] OR Arunachal Pradesh [tiab] OR Assam [tiab] OR Bihar[tiab] OR Chandigarh [tiab] OR Chattisgarh[tiab] OR Dadra and Nagar Haveli[tiab] OR Daman and Diu[tiab] OR Delhi [tiab] OR Goa [tiab] OR Gujarat [tiab] OR Haryana [tiab] OR Himachal Pradesh [tiab] OR Jammu and Kashmir [tiab] OR Jharkhand [tiab] OR Karnataka[tiab] OR Lakshadweep[tiab] OR Madhya Pradesh[tiab] OR Maharashtra[tiab] OR Manipur[tiab] ORMeghalaya[tiab] OR Mizoram [tiab]OR Nagaland[tiab] OR Odisha[tiab] OR Puducherry [tiab] OR Punjab[tiab] OR Rajasthan[tiab] OR Sikkim [tiab] OR Telangana[tiab] OR Tripura [tiab] OR Uttar Pradesh [tiab] OR West Bengal[tiab]))))))))))))))))))))))))))))))))))) AND Dengue[MeSH Major Topic]
Mortality	((Mortality[Title/Abstract]) AND (((((((((((((((((((((((((((((((((((India [Title/Abstract] OR Kerala[tiab] OR Tamil Nadu [tiab] OR Andaman and Nicobar[tiab] OR Arunachal Pradesh [tiab] OR Assam [tiab] OR Bihar[tiab] OR Chandigarh [tiab] OR Chattisgarh[tiab] OR Dadra and Nagar Haveli[tiab] OR Daman and Diu[tiab] OR Delhi [tiab] OR Goa [tiab] OR Gujarat [tiab] OR Haryana [tiab] OR Himachal Pradesh [tiab] OR Jammu and Kashmir [tiab] OR Jharkhand [tiab] OR Karnataka[tiab] OR Lakshadweep[tiab] OR Madhya Pradesh[tiab] OR Maharashtra[tiab] OR Manipur[tiab] ORMeghalaya[tiab] OR Mizoram [tiab]OR Nagaland[tiab] OR Odisha[tiab] OR Puducherry [tiab] OR Punjab[tiab] OR Rajasthan[tiab] OR Sikkim [tiab] OR Telangana[tiab] OR Tripura [tiab] OR Uttar Pradesh [tiab] OR West Bengal[tiab]))))))))))))))))))))))))))))))))))) AND Dengue[MeSH Major Topic]
Risk factors	((Riskfators[Title/Abstract]) AND ((((((((((((((((((India [Title/Abstract] OR Kerala[tiab] OR Tamil Nadu[tiab] OR Andaman and Nicobar[tiab] OR Arunachal Pradesh [tiab] OR Assam [tiab] OR Bihar[tiab] OR Chandigarh[tiab] OR Chattisgarh[tiab] OR Dadra and Nagar Haveli[tiab] OR Daman and Diu[tiab] OR Delhi [tiab] ORGoa [tiab] OR Gujarat [tiab] OR Haryana [tiab] OR Himachal Pradesh [tiab] OR Jammu and Kashmir [tiab] OR Jharkhand [tiab] OR Karnataka[tiab] OR Lakshadweep[tiab] OR Madhya Pradesh[tiab] OR Maharashtra[tiab] OR Manipur[tiab] ORMeghalaya[tiab] OR Mizoram [tiab]OR Nagaland[tiab] OR Odisha[tiab] OR Puducherry [tiab] OR Punjab[tiab] OR Rajasthan[tiab] OR Sikkim [tiab] OR Telangana[tiab] OR Tripura [tiab] OR Uttar Pradesh [tiab] OR West Bengal[tiab])))))))))))))))))))) AND Dengue[MeSH Major Topic]
Cost of illness	((Cost of illness[Title/Abstract]) AND ((((((((((((((((India [Title/Abstract] OR Kerala[tiab] OR Tamil Nadu [tiab] OR Andaman and Nicobar[tiab] OR Arunachal Pradesh [tiab] OR Assam [tiab] OR Bihar[tiab] OR Chandigarh [tiab] OR Chattisgarh[tiab] OR Dadra and Nagar Haveli[tiab] OR Daman and Diu[tiab] OR Delhi [tiab] OR Goa [tiab] OR Gujarat [tiab] OR Haryana [tiab] OR Himachal Pradesh [tiab] OR Jammu and Kashmir [tiab] OR Jharkhand [tiab] OR Karnataka[tiab] OR Lakshadweep[tiab] OR Madhya Pradesh[tiab] OR Maharashtra[tiab] OR Manipur[tiab] OR Meghalaya[tiab] OR Mizoram [tiab]OR Nagaland[tiab] OR Odisha[tiab] OR Puducherry [tiab] OR Punjab[tiab] OR Rajasthan[tiab] OR Sikkim [tiab] OR Telangana[tiab] OR Tripura [tiab] OR Uttar Pradesh [tiab] OR WestBengal[tiab])))))))))))) AND Dengue[MeSH Major Topic]

Economic burden	((Economic burden[Title/Abstract]) AN((((((((((((((((((((((((India [Title/Abstract] OR Kerala[tiab] OR Tamil Nadu [tiab] OR Andaman and Nicobar[tiab] OR Arunachal Pradesh [tiab] OR Assam [tiab] OR Bihar[tiab] OR Chandigarh [tiab] OR Chattisgarh[tiab] OR Dadra and Nagar Haveli[tiab] OR Daman and Diu[tiab] OR Delhi [tiab] OR Goa [tiab] OR Gujarat [tiab] OR Haryana [tiab] OR Himachal Pradesh [tiab] OR Jammu and Kashmir [tiab] OR Jharkhand [tiab] OR Karnataka[tiab] OR Lakshadweep[tiab] OR Madhya Pradesh[tiab] OR Maharashtra[tiab] OR Manipur[tiab] OR Meghalaya[tiab] OR Mizoram [tiab]OR Nagaland[tiab] OR Odisha[tiab] OR Puducherry [tiab] OR Punjab[tiab] OR Rajasthan[tiab] OR Sikkim [tiab] OR Telangana[tiab] OR Tripura [tiab] OR Uttar Pradesh [tiab] OR West Bengal[tiab]))))))))))))))))))))))))))))))))))) AND Dengue[MeSH Major Topic]
Burden of disease	((Burden of disease[Title/Abstract]) AND ((((((((((((((((((((((((((((((((((India [Title/Abstract] OR Kerala[tiab] OR Tamil Nadu [tiab] OR Andaman and Nicobar[tiab] OR Arunachal Pradesh [tiab] OR Assam [tiab] OR Bihar[tiab] OR Chandigarh [tiab] OR Chattisgarh[tiab] OR Dadra and Nagar Haveli[tiab] OR Daman and Diu[tiab] OR Delhi [tiab] OR Goa [tiab] OR Gujarat [tiab] OR Haryana [tiab] OR Himachal Pradesh [tiab] OR Jammu and Kashmir [tiab] OR Jharkhand [tiab] OR Karnataka[tiab] OR Lakshadweep[tiab] OR Madhya Pradesh[tiab] OR Maharashtra[tiab] OR Manipur[tiab] OR Meghalaya[tiab] OR Mizoram [tiab]OR Nagaland[tiab] OR Odisha[tiab] OR Puducherry [tiab] OR Punjab[tiab] OR Rajasthan[tiab] OR Sikkim [tiab] OR Telangana[tiab] OR Tripura [tiab] OR Uttar Pradesh [tiab] OR West Bengal[tiab])))))))))))))))))))))))))))))))))) AND Dengue[MeSH Major Topic]
DALY	((DALY[Title/Abstract]) AND ((((((((((((((((((((((((((((((((((India [Title/Abstract] OR Kerala[tiab] OR Tamil Nadu [tiab] OR Andaman and Nicobar[tiab] OR Arunachal Pradesh [tiab] OR Assam [tiab] OR Bihar[tiab] OR Chandigarh [tiab] OR Chattisgarh[tiab] OR Dadra and Nagar Haveli[tiab] OR Daman and Diu[tiab] OR Delhi [tiab] OR Goa [tiab] OR Gujarat [tiab] OR Haryana [tiab] OR Himachal Pradesh [tiab] OR Jammu and Kashmir [tiab] OR Jharkhand [tiab] OR Karnataka[tiab] OR Lakshadweep[tiab] OR Madhya Pradesh[tiab] OR Maharashtra[tiab] OR Manipur[tiab] ORMeghalaya[tiab] OR Mizoram [tiab]OR Nagaland[tiab] OR Odisha[tiab] OR Puducherry [tiab] OR Punjab[tiab] OR Rajasthan[tiab] OR Sikkim [tiab] OR Telangana[tiab] OR Tripura [tiab] OR Uttar Pradesh [tiab] OR West Bengal[tiab])))))))))))))))))))))))))))))))))) AND Dengue[MeSH Major Topic]
