# Supplementary material for: Dengue infection in India: A systematic review and meta-analysis
Source: PLoS Negl Trop Dis. 2018 Jul 16;12(7):e0006618. doi: 10.1371/journal.pntd.0006618 (PMC6078327; doi:10.1371/journal.pntd.0006618)
Supplement: S2 Appendix — (PDF) [file pntd.0006618.s002.pdf]

Critical appraisal checklist for Quality Assessment of Studies

S.no	Studies to address	Criteria items and the adopted score
MAJOR CRITERIA		
1	Inclusion of study participants- Whether case definition of dengue is mentioned	A. NVBDCP/WHO case definition = 1<br>B. Fever case definition/ Suspected / clinical dengue = 0.5<br>C. No case definition/ Unclear = 0
2	Whether any sampling method for inclusion of patients is adopted in the study?	A. Included all/ Systematically/ Randomly = 1<br>B. Unknown/ Unclear = 0
3	Measurement of disease condition	A. IgM/NS1/PCR = 1<br>B. Serological = 0.5<br>C. No lab test/Unclear = 0
4	Whether total number tested is mentioned in the paper?	A. Yes = 1<br>B. Unclear/No = 0
MINOR CRITERIA		
1	Whether age distribution of cases is given?	A. Both in tested and positive = 1<br>B. Only in tested or positive = 0.5<br>C. Not given/Unlcear = 0
2	Whether study is not limited any speciality setting?	A. Yes = 1<br>B. No/ Unclear = 0
3	Whether CFR is reported?	A. Yes = 1<br>B. No/ Unclear = 0

Overall risk of bias assessment

QUALITY	RISK OF BIAS	CRITERIA
HIGH	LOW	ALL MAJOR = 1 + ALL MINOR = 0 OR ONLY ONE MINOR = 0
MODERATE	MODERATE	2 MAJOR = 1 + LESS THAN ONE MINOR = 0

LOW	HIGH	2 MAJOR = 0 + TWO MINOR = 0
VERY LOW	VERY HIGH	NO MAJOR = 1 + NO MINOR = 1
