# Supplementary material for: Dengue infection in India: A systematic review and meta-analysis
Source: PLoS Negl Trop Dis. 2018 Jul 16;12(7):e0006618. doi: 10.1371/journal.pntd.0006618 (PMC6078327; doi:10.1371/journal.pntd.0006618)

A1. Sensitivity analysis of laboratory confirmed dengue infection in Hospital based studies

Author(s) and Year(Study Omitted)	Dengue Proportion (%) [95% CI]
Chaturvedi et al, 1970 [44]	33.7 [29.9 , 37.6]
Mohan et al, 2000 [85]	33.4 [29.7 , 37.3]
Gomber et al, 2001 [53]	33.2 [29.6 , 37.1]
Vajpayee et al, 1999 [127]	33.6 [29.9 , 37.6]
Singh et al, 2000 [115]	33.2 [29.6 , 37.1]
Chakravarti et al, 2002* [37]	33.9 [30.2 , 37.8]
Chatterjee et al, 2013* [43]	33.6 [29.9 , 37.6]
Kumaria et al, 2003* [75]	33.9 [30.1 , 37.8]
Vijayakumar et al, 2005* [130]	33.6 [29.9 , 37.6]
Karande et al, 2005 [68]	33.6 [29.9 , 37.5]
Shivbalan et al, 2004* [113]	33.2 [29.6 , 37.0]
Bhattacharya et al, 2004* [33]	33.5 [29.8 , 37.4]
Faridi et al, 2008 [51]	33.4 [29.7 , 37.2]
Gupta et al, 2005 [57]	33.4 [29.7 , 37.4]
Gupta et al, 2006* [56]	33.5 [29.8 , 37.4]
Kumaria et al, 2010* [76]	33.7 [29.9 , 37.6]
Chakravarti et al, 2012* [38]	33.6 [29.9 , 37.6]
Hati et al, 2006 [60]	33.5 [29.8 , 37.4]
Lal et al, 2007 [77]	33.5 [29.8 , 37.5]
Priyadarshni et al, 2010 [98]	33.4 [29.7 , 37.3]
Saxena et al, 2006 [107]	33.6 [29.9 , 37.6]
Tripathi et al, 2008* [125]	33.7 [30.0 , 37.6]
Zaki et al, 2010 [133]	33.8 [30.1 , 37.7]
Chandralekha et al, 2008 [41]	33.5 [29.8 , 37.4]
Chhina et al, 2009 [45]	33.8 [30.1 , 37.8]
Hati et al, 2009* [61]	33.8 [30.0 , 37.7]
Kulkarni et al, 2010 [71]	33.3 [29.6 , 37.1]
Kumar et al, 2008* [74]	33.7 [30.0 , 37.7]
Ukey et al, 2010* [126]	33.6 [29.9 , 37.5]
Gunasekaran et al, 2011* [55]	33.5 [29.8 , 37.4]
Agarwal et al, 2010 [23]	33.4 [29.7 , 37.3]
Bhaskar et al, 2010* [32]	33.5 [29.8 , 37.4]
Chakravarti et al, 2010 [36]	33.7 [30.0 , 37.6]
Chakravarti et al, 2013* [39]	33.9 [30.2 , 37.8]
Rashmi Kumar et al, 2008 [102]	33.2 [29.6 , 37.0]
Chrispal et al, 2010* [46]	33.9 [30.3 , 37.8]
Garg et al, 2011* [52]	33.7 [30.0 , 37.7]
Hati et al, 2012* [62]	33.7 [30.0 , 37.7]
Kanungo et al, 2009 [67]	33.9 [30.2 , 37.8]
Kumar et al, 2012* [72]	33.8 [30.1 , 37.7]
Taraphdar et al, 2012* [122]	33.9 [30.2 , 37.8]
Sharma et al, 2012* [112]	33.7 [30.0 , 37.7]
Duthade et al, 2011 [48]	33.7 [30.0 , 37.6]
Kumar et al, 2013* [73]	33.6 [29.9 , 37.5]
Pandey et al, 2012* [93]	33.4 [29.7 , 37.3]
Raut et al, 2012* [103]	33.6 [29.9 , 37.6]
Tank Arun et al, 2012* [121]	33.6 [29.9 , 37.5]
Barde et al, 2012 [29]	33.7 [30.0 , 37.6]
Dutta et al, 2012* [50]	33.6 [29.9 , 37.5]
Kalappanvar et al, 2013* [66]	33.7 [30.0 , 37.6]
Karoli et al, 2012 [69]	33.5 [29.8 , 37.5]
Manakkadan et al, 2013* [80]	33.7 [30.0 , 37.6]
Mehta et al, 2014* [81]	33.6 [29.9 , 37.6]
Shaikh et al, 2015 [109]	33.5 [29.7 , 37.4]
Sood et al, 2013* [119]	33.7 [30.0 , 37.7]
Taraphdar et al, 2012 [123]	33.7 [30.0 , 37.7]
Anand et al, 2016 [27]	33.1 [29.5 , 37.0]
Bandyopadhyay et al, 2013* [28]	33.7 [29.9 , 37.6]
Bhuvaneswari et al, 2011 [35]	33.5 [29.8 , 37.5]
Gupta et al, 2012* [58]	33.7 [29.9 , 37.6]
Henna et al, 2014* [63]	33.6 [29.8 , 37.5]
Lata et al, 2017 [79]	33.5 [29.7 , 37.4]
Mishra et al, 2015* [82]	33.6 [29.9 , 37.5]
Nujum et al, 2014 [89]	33.7 [30.0 , 37.7]
Prasad et al, 2013 [97]	33.6 [29.9 , 37.6]
Padhi et al, 2014* [90]	33.7 [30.0 , 37.6]
Sushi et al, 2014 [120]	33.9 [30.2 , 37.8]
Venkatasubramani et al, 2015* [134]	33.8 [30.1 , 37.7]
Amudhan et al, 2015* [26]	33.7 [29.9 , 37.6]
Barde et al, 2014* [30]	33.8 [30.1 , 37.7]
Neeraja et al, 2014* [86]	33.4 [29.7 , 37.2]
Prakash et al, 2015* [96]	33.7 [30.0 , 37.6]
Padmapriya et al, 2017* [91]	33.7 [30.0 , 37.7]
Patankar et al, 2014 [94]	33.7 [30.0 , 37.6]
Saraswathy et al, 2013* [105]	33.5 [29.8 , 37.4]
Singla et al, 2015* [118]	33.9 [30.2 , 37.8]
Rao et al, 2013 [101]	33.5 [29.8 , 37.5]
Vinodkumar et al, 2014* [131]	33.4 [29.7 , 37.3]
Yogeesha et al, 2014 [132]	33.5 [29.8 , 37.5]
Abhilash et al, 2016* [21]	33.6 [29.9 , 37.6]
Ahmad et al, 2016* [25]	33.6 [29.9 , 37.5]
Bhattacharya et al, 2017 [34]	33.2 [29.6 , 37.0]
Chakravarti et al, 2014 [40]	33.5 [29.8 , 37.5]
Gopal et al, 2016 [54]	33.5 [29.8 , 37.4]
Henna et al, 2014* [63]	33.7 [29.9 , 37.6]
Mistry et al, 2015 [83]	33.5 [29.8 , 37.5]
Prudhivi et al, 2014 [99]	33.7 [29.9 , 37.6]
Singh et al, 2014 [117]	33.3 [29.6 , 37.1]
Rao et al, 2016 [100]	33.5 [29.8 , 37.5]
Saini et al, 2013* [104]	33.6 [29.9 , 37.6]
Saswat et al, 2015 [106]	33.6 [29.8 , 37.5]
Pothapregada et al, 2016* [95]	33.3 [29.6 , 37.2]
Sharma et al, 2014 [111]	33.7 [30.0 , 37.6]
Afreen et al, 2015* [22]	33.3 [29.6 , 37.2]
Barua et al, 2016 [31]	33.3 [29.6 , 37.2]
Deshkar et al, 2017* [47]	33.7 [29.9 , 37.6]
Duthade et al, 2015 [49]	33.6 [29.9 , 37.6]
Gusani et al, 2017 [59]	33.5 [29.8 , 37.4]
Pai Jakribettu et al, 2015* [92]	33.6 [29.8 , 37.5]
Kaup et al, 2014* [70]	33.7 [30.0 , 37.6]
Nikam et al, 2015 [87]	33.6 [29.9 , 37.6]
Tazeen et al, 2017 [124]	33.2 [29.6 , 37.0]
Vakrani et al, 2017* [128]	33.3 [29.6 , 37.1]
Ahir et al, 2016* [24]	33.8 [30.1 , 37.8]
Changal et al, 2016 [42]	33.4 [29.7 , 37.4]
Islam et al, 2016 [64]	33.6 [29.9 , 37.6]
Lall et al, 2016 [78]	33.7 [30.0 , 37.7]
Mital et al, 2016 [84]	33.3 [29.6 , 37.2]
Shabnum et al, 2017 [108]	33.5 [29.8 , 37.4]
Siddiqui et al, 2016 [114]	33.6 [29.9 , 37.5]
Sharma et al, 2016 [110]	33.6 [29.9 , 37.6]
Joshua et al, 2016* [65]	33.5 [29.7 , 37.4]
Singh et al, 2016* [116]	33.4 [29.7 , 37.3]
Nisarta et al, 2016* [88]	33.7 [29.9 , 37.6]
Verma et al, 2016 [129]	33.7 [29.9 , 37.6]

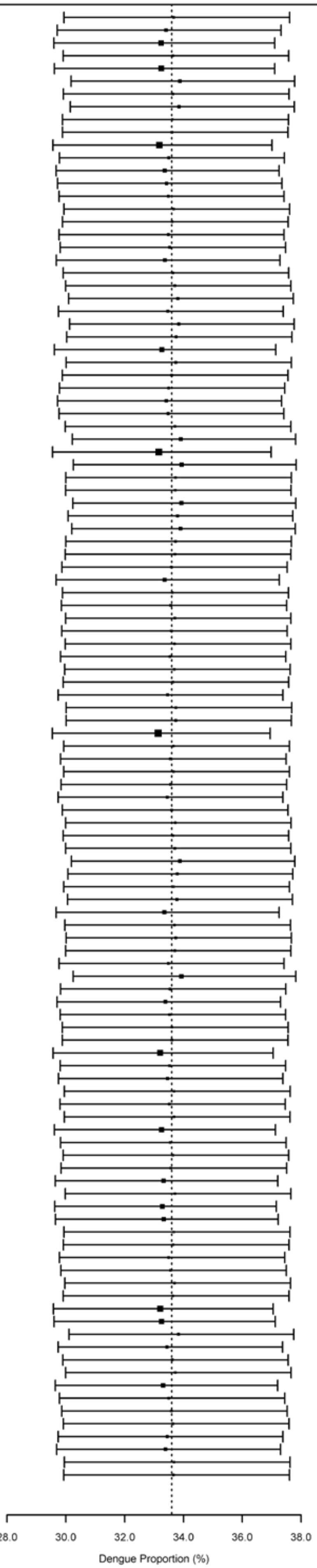

A2. Funnel plot of laboratory confirmed dengue infection in Hospital based studies

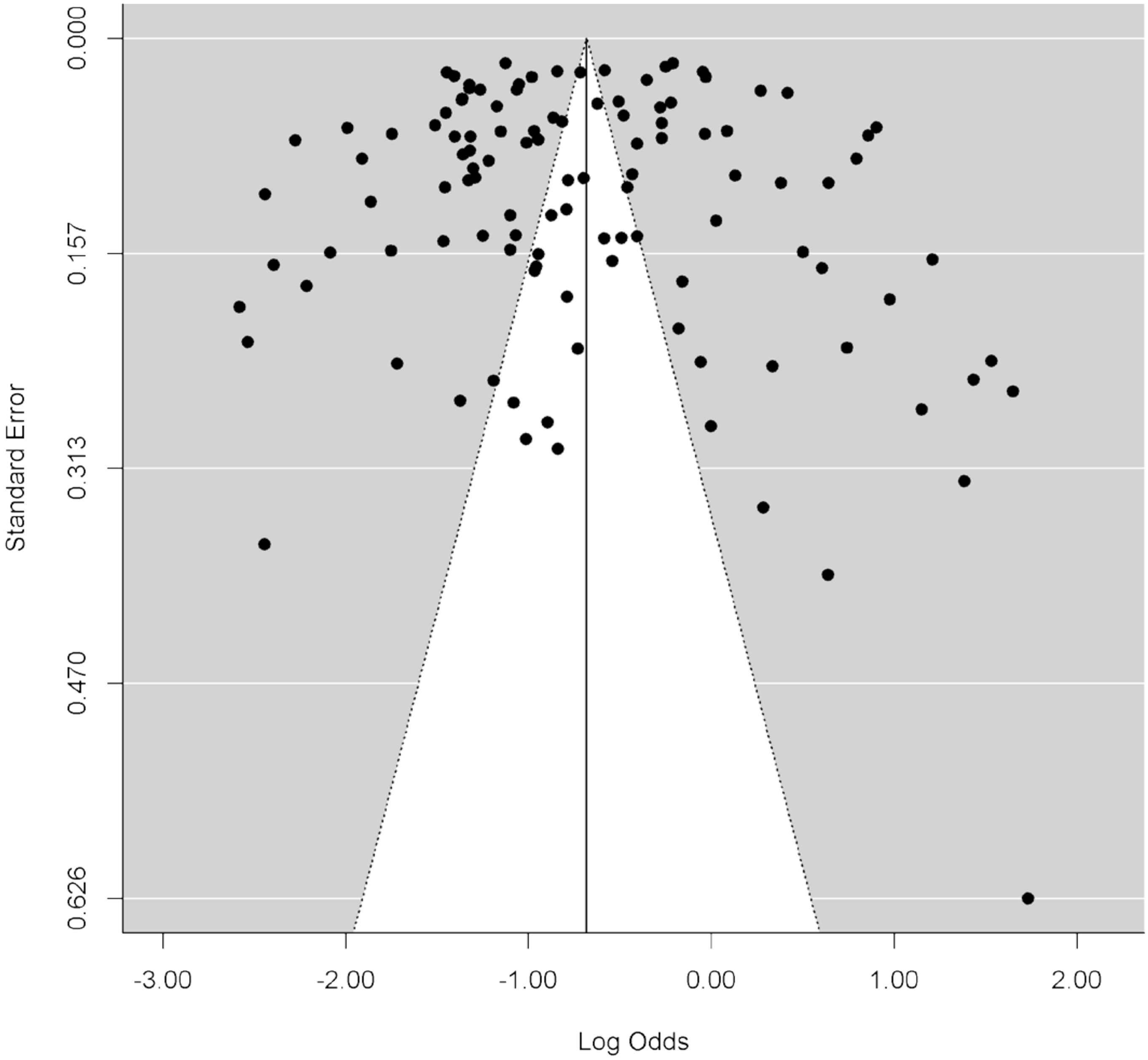

B1. Sensitivity analysis of laboratory confirmed dengue infection in Hospital based studies during outbreak or outbreaks

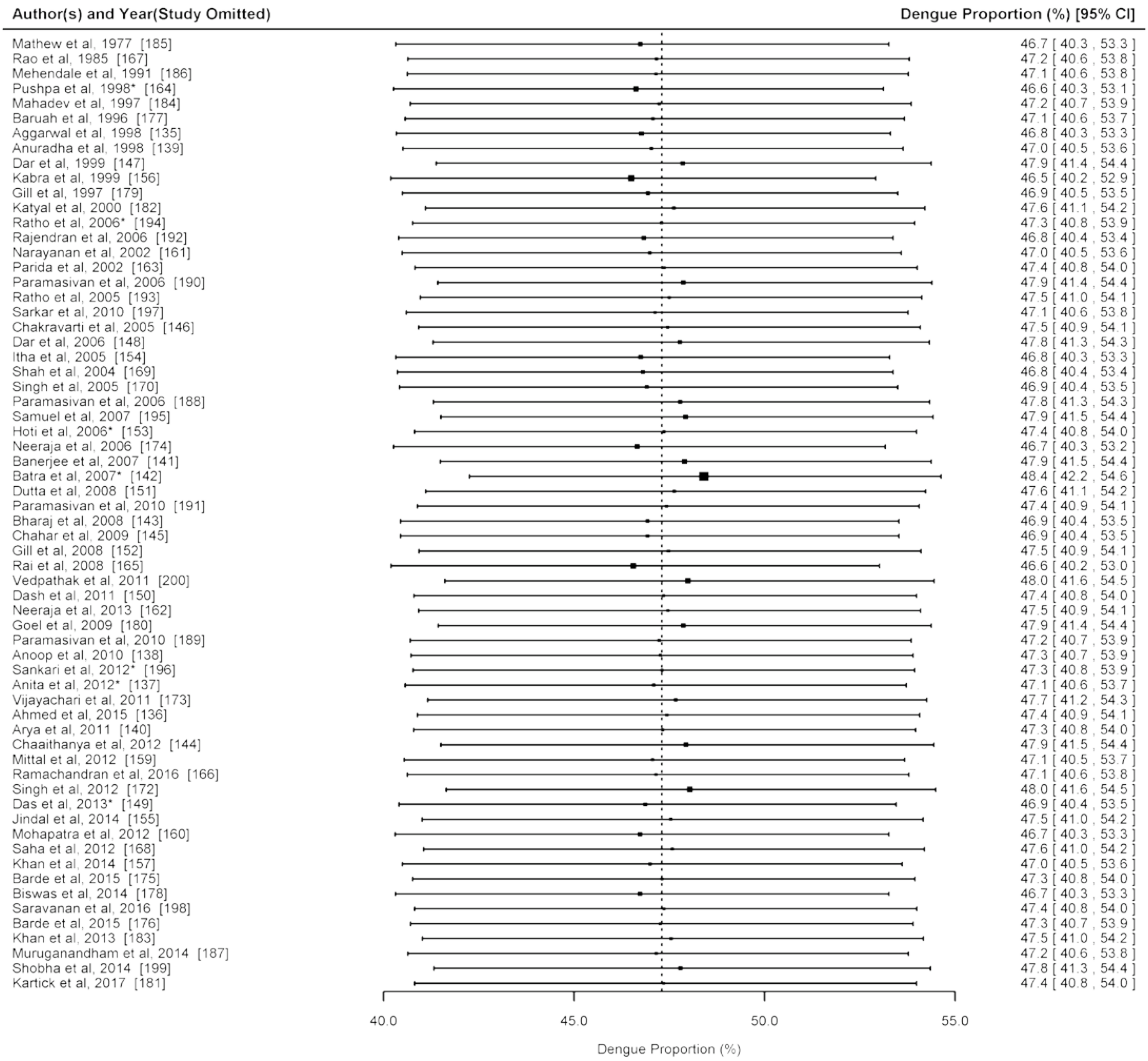

B2. Funnel plot of laboratory confirmed dengue infection in Hospital based studies during outbreak or outbreaks

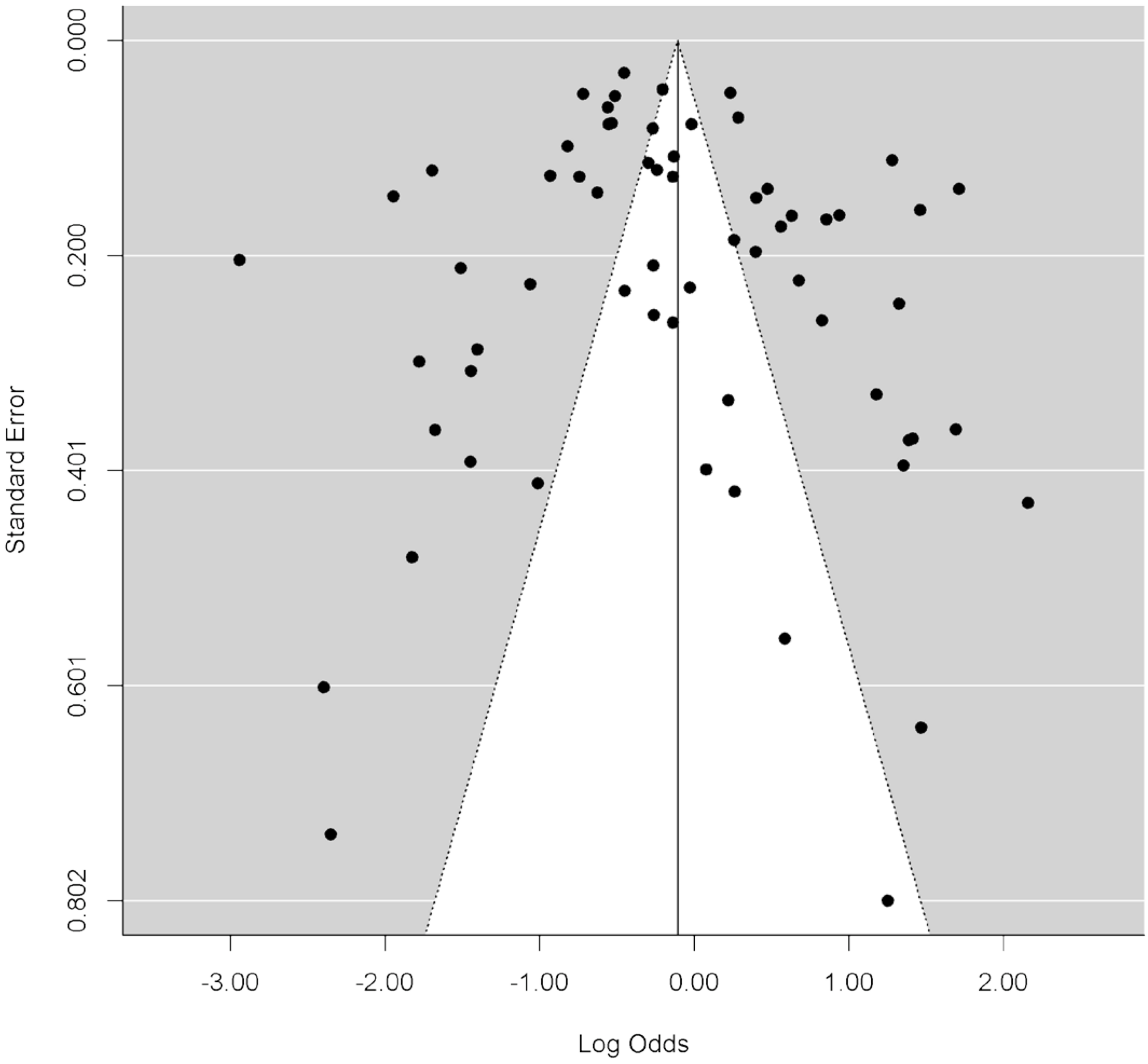

D1. Sensitivity analysis of studies reporting Seroprevalence of Dengue

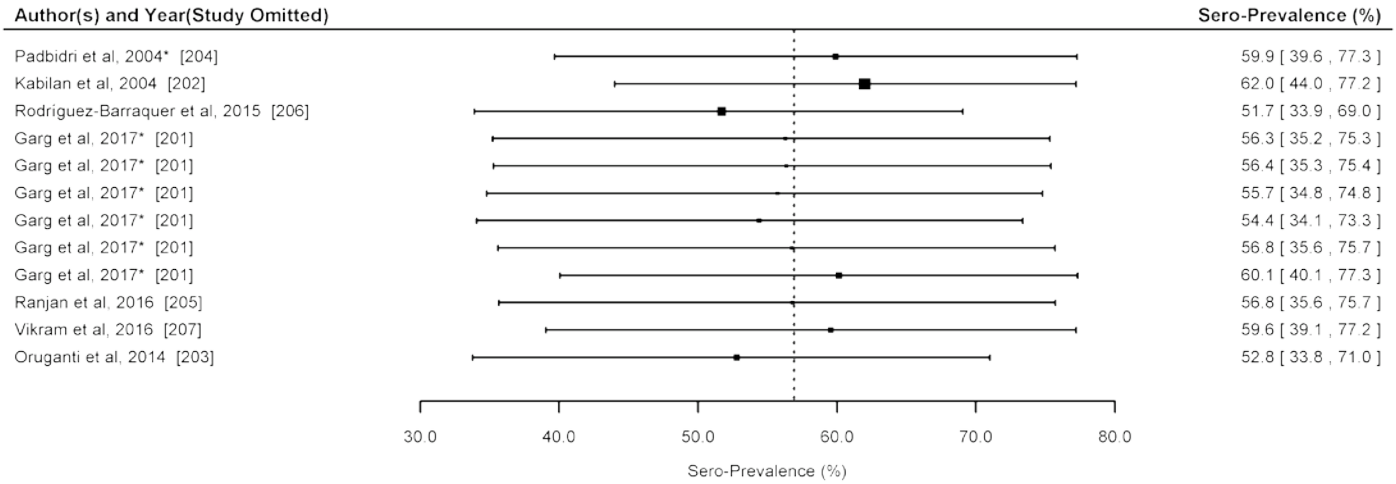

D2. Funnel plot of studies reporting Seroprevalence of Dengue

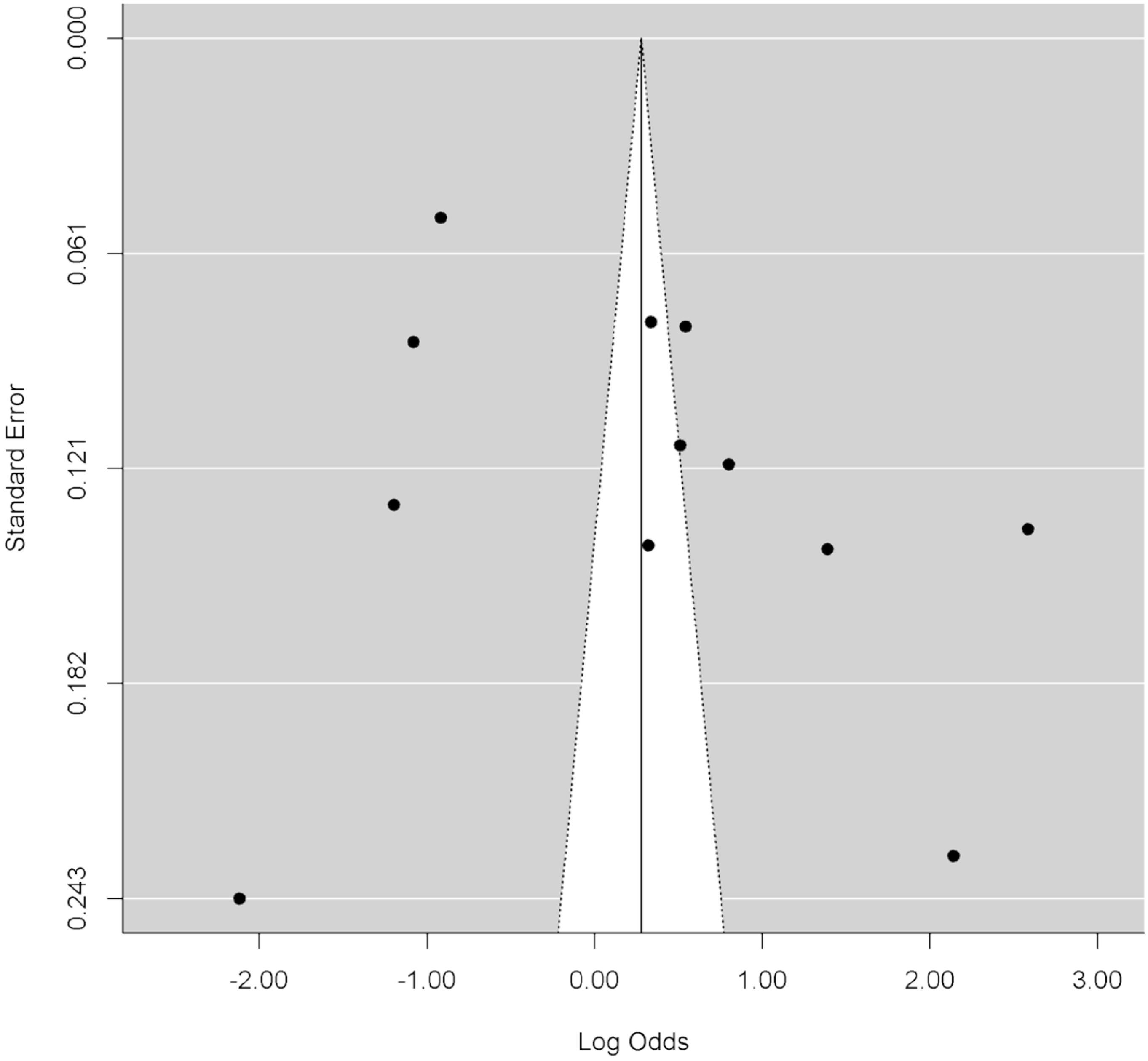

E1. Sensitivity analysis of studies reporting Case fatality ratio of Dengue

Author(s) and Year (study Omitted)	Case Fatality Ratio (%) [95% CI]
Kabra et al, 1992 [220]	2.5 [-0.2 , 5.3]
Pushpa et al, 1998* [164]	2.5 [-0.2 , 5.2]
Agarwal et al, 1999 [208]	2.5 [-0.2 , 5.3]
Singh et al, 2000 [115]	2.5 [-0.2 , 5.3]
Narayanan et al, 2002 [161]	2.6 [-0.2 , 5.3]
Kamath et al, 2006* [222]	2.5 [-0.2 , 5.3]
Itha et al, 2005 [154]	2.5 [-0.2 , 5.2]
Samuel et al, 2007 [195]	2.6 [-0.2 , 5.3]
Shah et al, 2004 [169]	2.5 [-0.2 , 5.3]
Singh et al, 2005 [170]	2.6 [-0.2 , 5.3]
Daniel et al, 2005 [212]	2.6 [-0.2 , 5.3]
Ratageri et al, 2005* [239]	2.6 [-0.2 , 5.3]
Hati et al, 2006 [60]	2.7 [-0.1 , 5.5]
Priyadarshni et al, 2010 [98]	2.6 [-0.2 , 5.4]
Zaki et al, 2010 [133]	2.6 [-0.2 , 5.3]
Kumar et al, 2010* [209]	2.6 [-0.2 , 5.3]
Chhina et al, 2009 [45]	2.6 [-0.2 , 5.3]
Hati et al, 2009* [61]	2.7 [-0.1 , 5.5]
Rai et al, 2008 [165]	2.5 [-0.2 , 5.3]
Nandi et al, 2008* [238]	2.6 [-0.2 , 5.4]
Neeraja et al, 2013 [162]	2.6 [-0.2 , 5.3]
Agarwal et al, 2010 [23]	2.6 [-0.2 , 5.3]
Rashmi Kumar et al, 2008 [102]	2.5 [-0.2 , 5.3]
Chrispal et al, 2010* [46]	2.5 [-0.2 , 5.2]
Kumar et al, 2012* [72]	2.5 [-0.2 , 5.3]
Anita et al, 2012* [137]	2.6 [-0.2 , 5.4]
Duthade et al, 2011 [48]	2.6 [-0.2 , 5.3]
Giri et al, 2010* [215]	2.6 [-0.2 , 5.4]
Joshi et al, 2011* [218]	2.6 [-0.2 , 5.3]
Juneja et al, 2011* [219]	2.5 [-0.2 , 5.3]
Ahmed et al, 2015 [136]	2.7 [-0.1 , 5.5]
Kalappanvar et al, 2013* [66]	2.5 [-0.2 , 5.3]
Karoli et al, 2012 [69]	2.5 [-0.2 , 5.3]
Mittal et al, 2012 [159]	2.6 [-0.2 , 5.4]
Dhar et al, 2013 [236]	2.6 [-0.1 , 5.4]
Mukherjee et al, 2012 [237]	2.5 [-0.2 , 5.2]
Bhuvaneswari et al, 2011 [35]	2.6 [-0.2 , 5.4]
Lata et al, 2017 [79]	2.6 [-0.2 , 5.4]
Prasad et al, 2013 [97]	2.5 [-0.2 , 5.2]
Bhalla et al, 2014 [210]	2.6 [-0.1 , 5.4]
Nimmagadda et al, 2014* [226]	2.6 [-0.2 , 5.3]
Barde et al, 2015 [176]	2.6 [-0.2 , 5.3]
Neeraja et al, 2014* [86]	2.5 [-0.2 , 5.3]
Saravanan et al, 2016 [198]	2.6 [-0.2 , 5.3]
Kale et al, 2014* [221]	2.6 [-0.2 , 5.4]
Chatterjee et al, 2014 [225]	2.5 [-0.2 , 5.3]
Sahu et al, 2014* [228]	2.6 [-0.2 , 5.4]
Abhilash et al, 2016* [21]	2.6 [-0.2 , 5.3]
Ahmad et al, 2016* [25]	2.5 [-0.2 , 5.3]
Bhattacharya et al, 2017 [34]	2.6 [-0.2 , 5.4]
Singh et al, 2014 [117]	2.6 [-0.2 , 5.4]
Rao et al, 2016 [100]	2.6 [-0.1 , 5.4]
Pothapregada et al, 2015* [95]	2.6 [-0.2 , 5.3]
Vakrani et al, 2017* [128]	2.6 [-0.2 , 5.4]
Sharma et al, 2014 [111]	2.6 [-0.2 , 5.4]
Deshmukh et al, 2014* [213]	2.5 [-0.2 , 5.3]
Deshwal et al, 2015 [214]	2.6 [-0.1 , 5.4]
Gupta et al, 2015 [216]	2.6 [-0.2 , 5.4]
Krishnamoorthy et al, 2017 [223]	2.6 [-0.2 , 5.3]
Pothapregada et al, 2015* [227]	2.6 [-0.2 , 5.3]
Sharma et al, 2015 [231]	2.6 [-0.2 , 5.3]
Verma et al, 2014 [234]	2.5 [-0.2 , 5.3]
Srividhya et al, 2017 [235]	2.6 [-0.2 , 5.4]
Barua et al, 2016 [31]	2.6 [-0.2 , 5.4]
Deshkar et al, 2017* [47]	2.6 [-0.2 , 5.4]
Duthade et al, 2015 [49]	2.6 [-0.2 , 5.3]
Pai Jakribettu et al, 2015* [92]	2.6 [-0.2 , 5.3]
Mishra et al, 2016* [233]	2.6 [-0.2 , 5.4]
Chhotala et al, 2016* [211]	2.5 [-0.2 , 5.3]
Jain et al, 2017 [217]	2.5 [-0.2 , 5.3]
Saroch et al, 2017 [229]	2.5 [-0.2 , 5.2]
Nagaram et al, 2017* [224]	2.5 [-0.2 , 5.3]
Sharma et al, 2016* [230]	2.6 [-0.2 , 5.4]
Sharma et al, 2016* [232]	2.6 [-0.2 , 5.4]

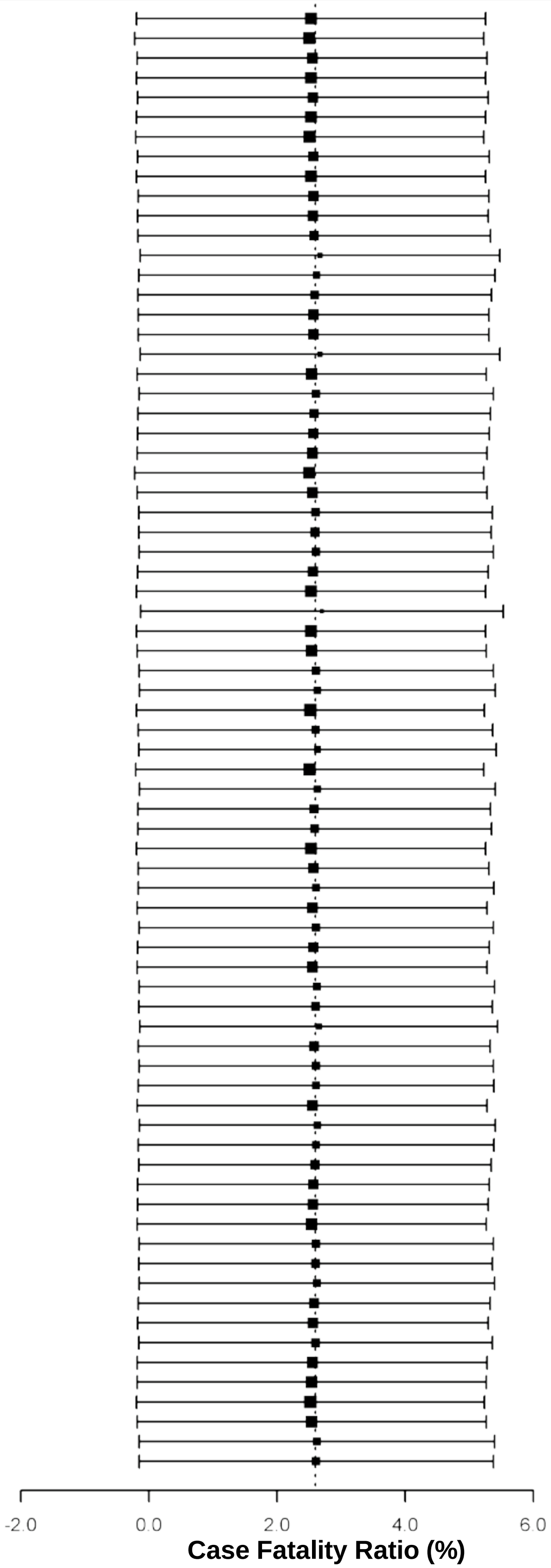

E2. Funnel plot of studies reporting Case fatality ratio of Dengue

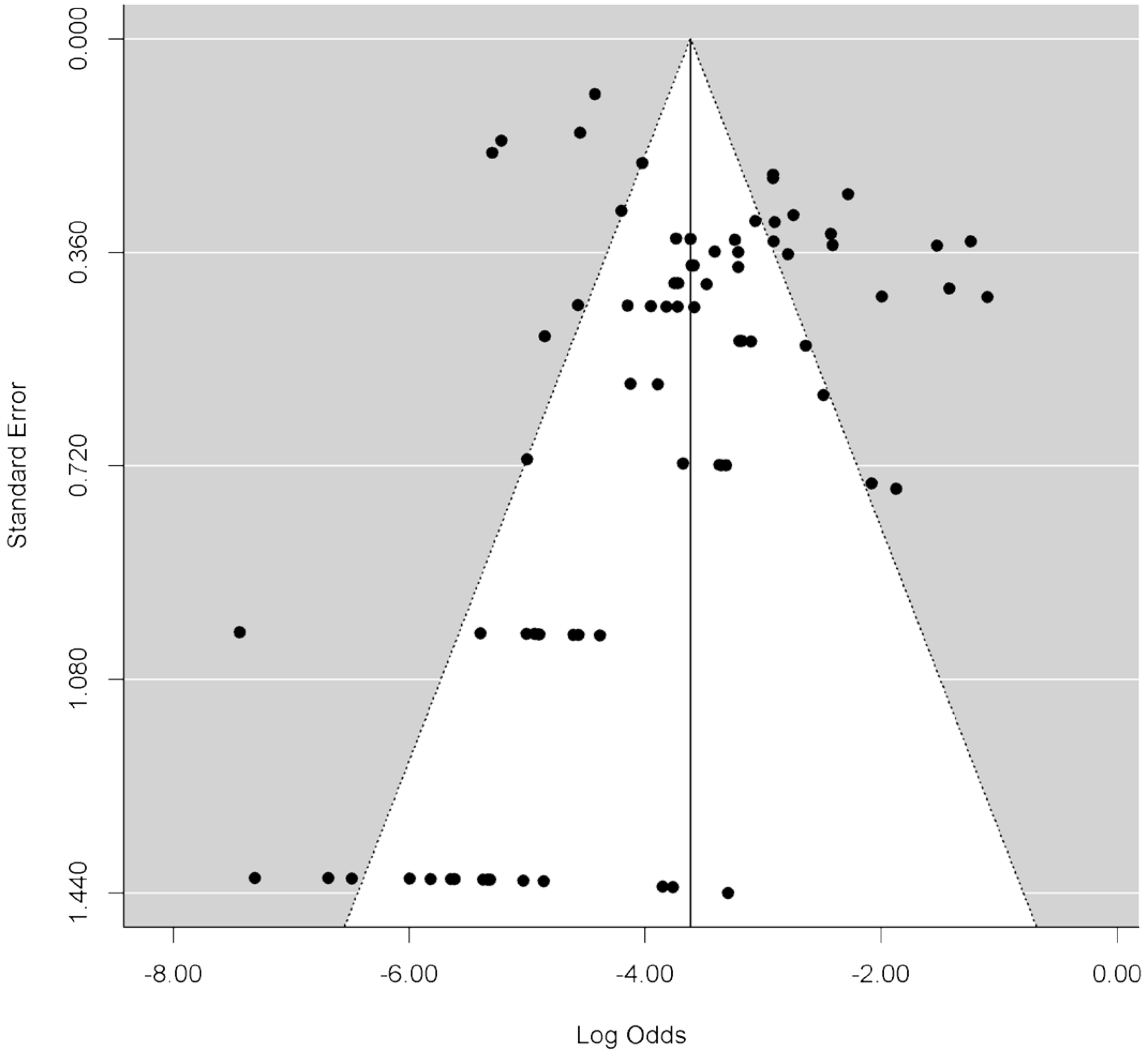

F1. Sensitivity analysis of studies reporting Secondary infection of Dengue

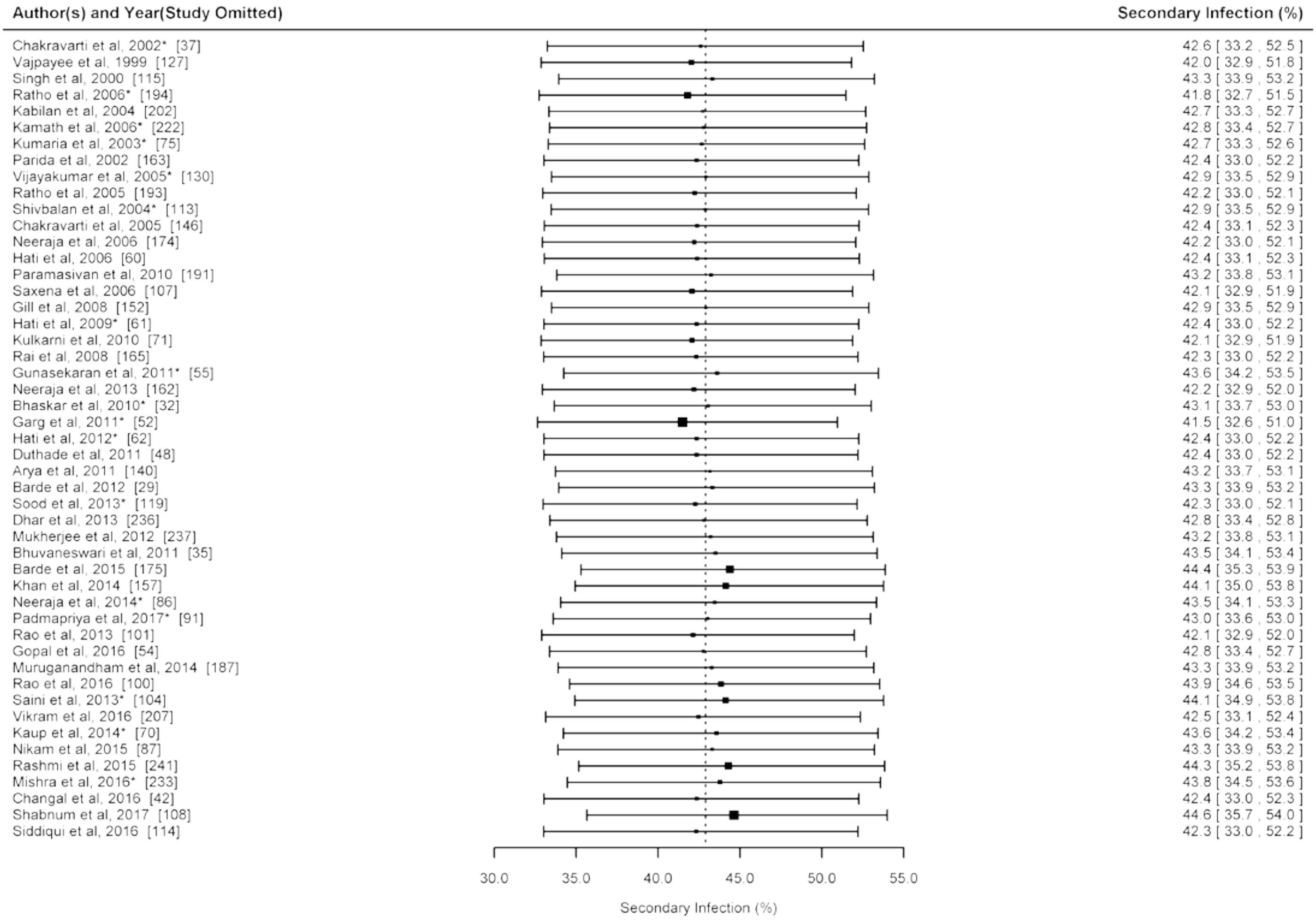

F2. Funnel plot of studies reporting Secondary infection of Dengue

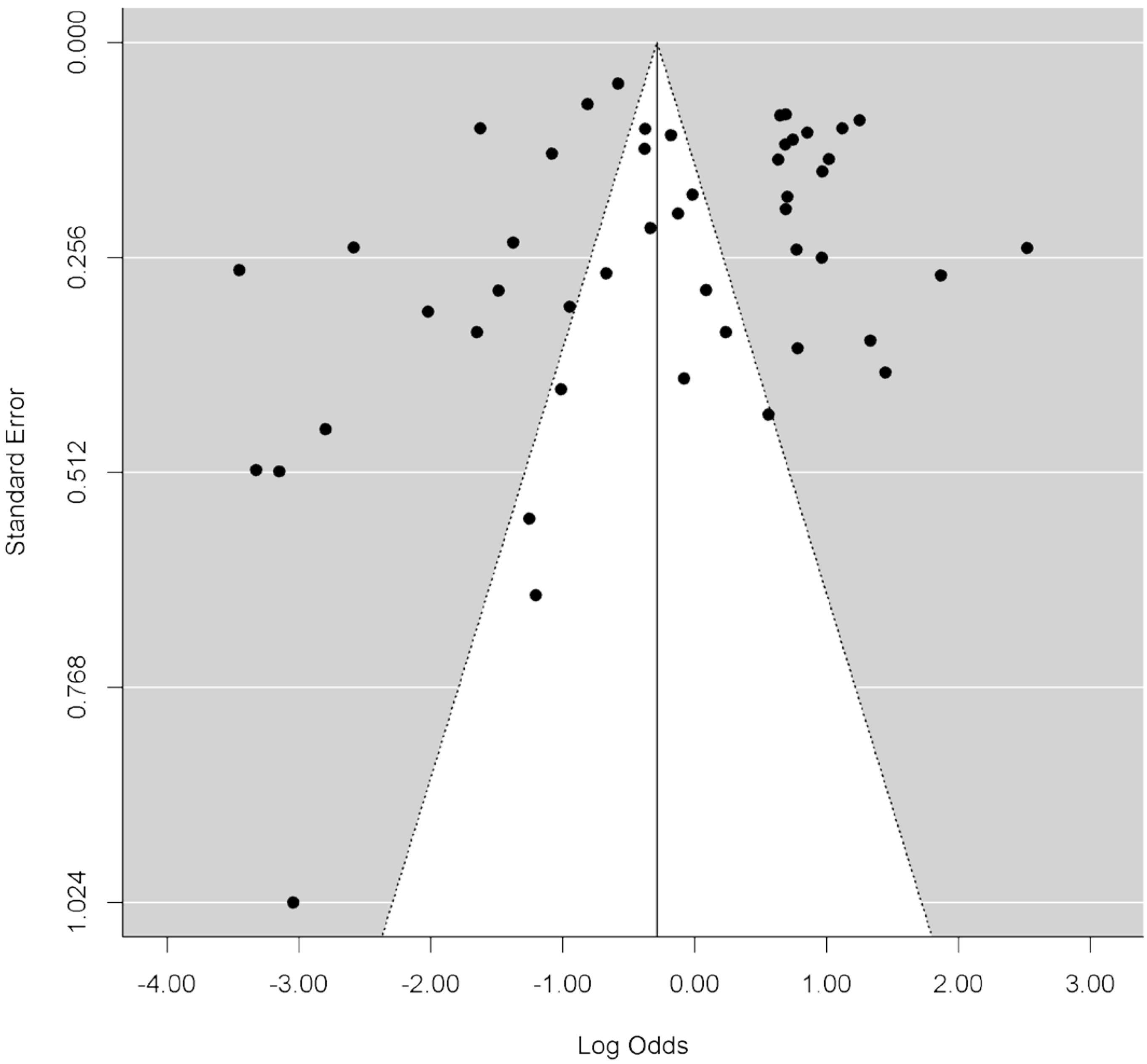

G1. Sensitivity analysis of studies reporting Severe Dengue fever

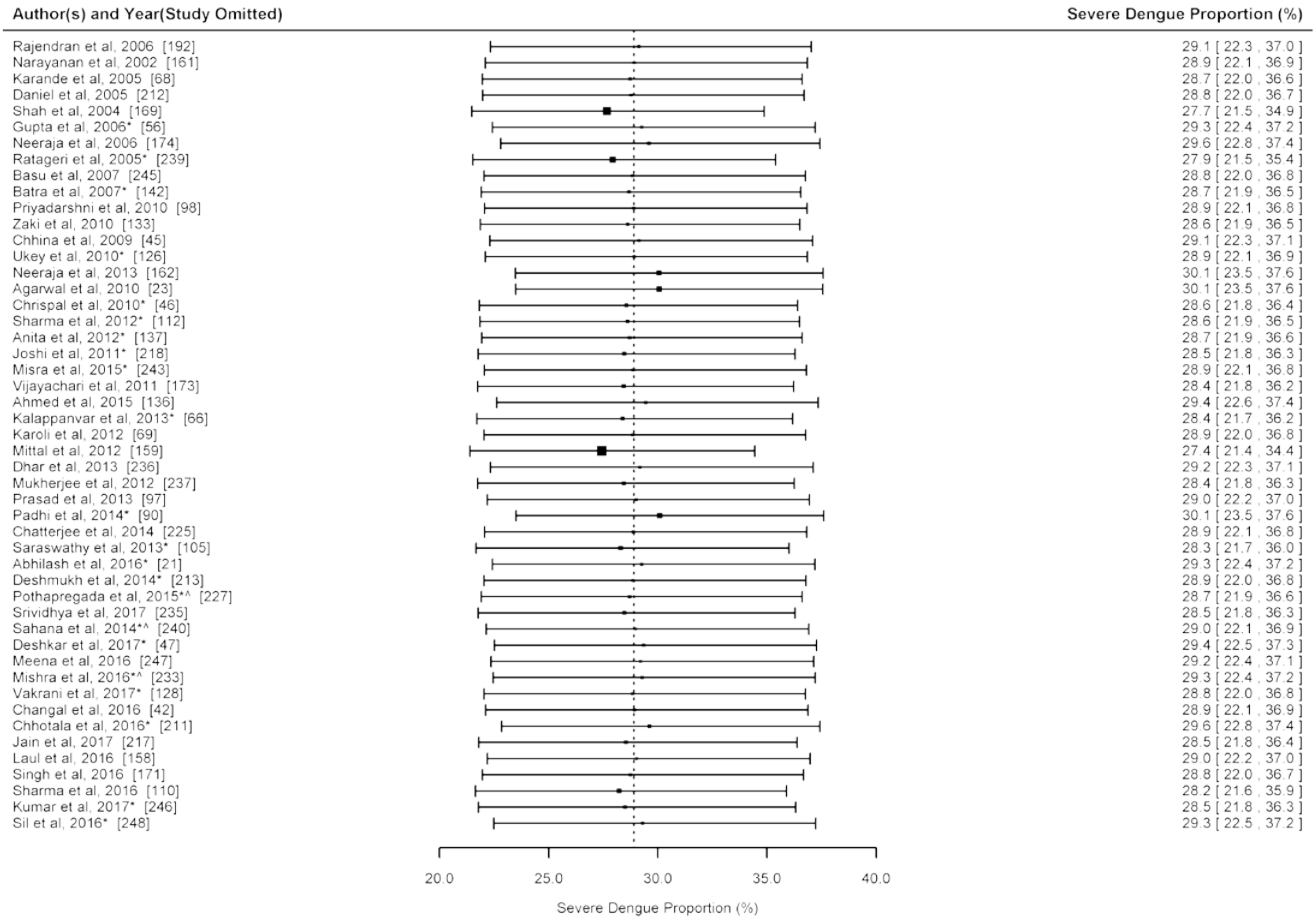

G2. Funnel plot of studies reporting Severe Dengue fever

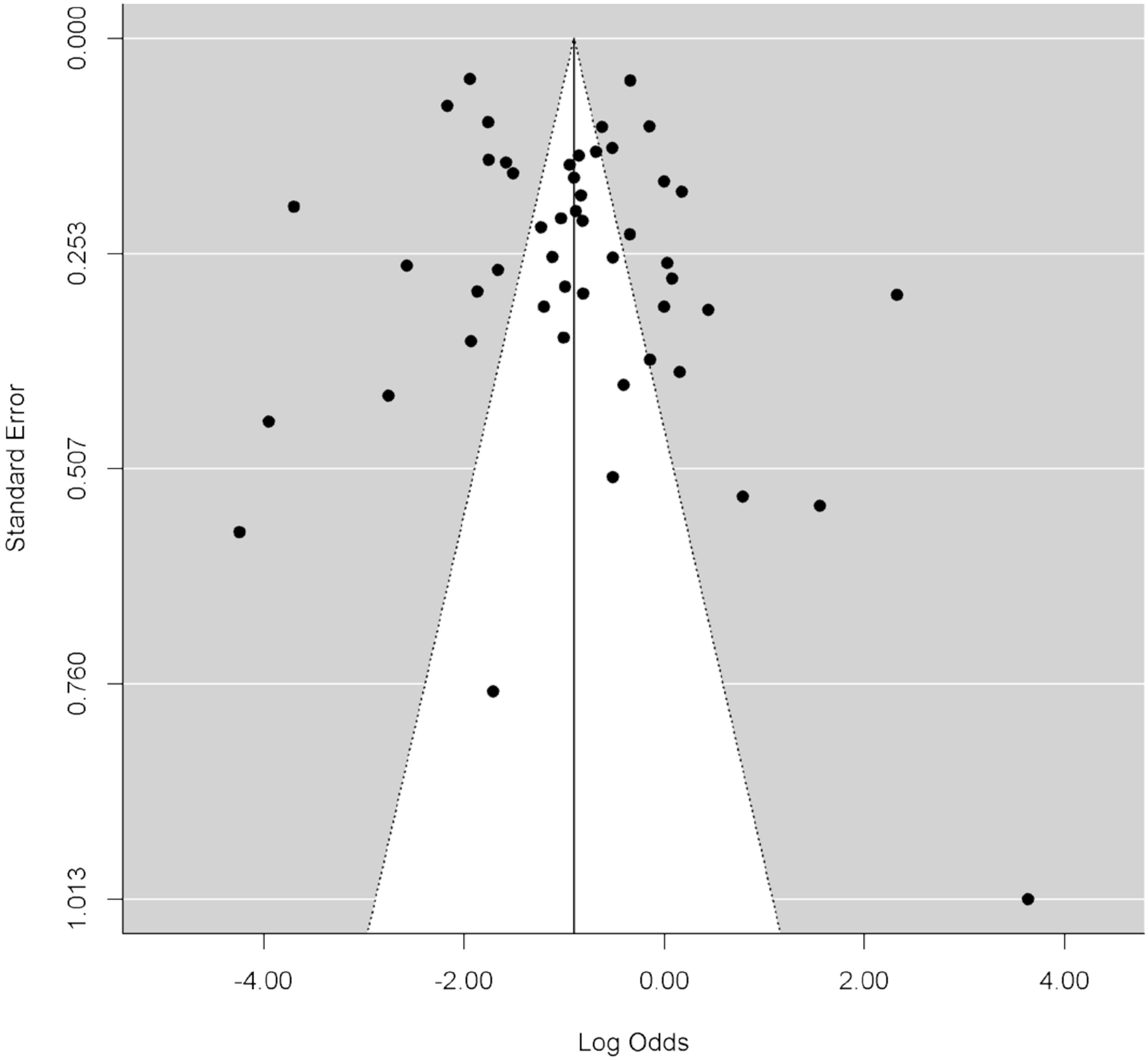

Supplement: S3 Appendix — (PDF) [file pntd.0006618.s003.pdf]
