# Supplementary material for: Dengue infection in India: A systematic review and meta-analysis
Source: PLoS Negl Trop Dis. 2018 Jul 16;12(7):e0006618. doi: 10.1371/journal.pntd.0006618 (PMC6078327; doi:10.1371/journal.pntd.0006618)

A. Prevalence of lab confirmed dengue in hospital based studies

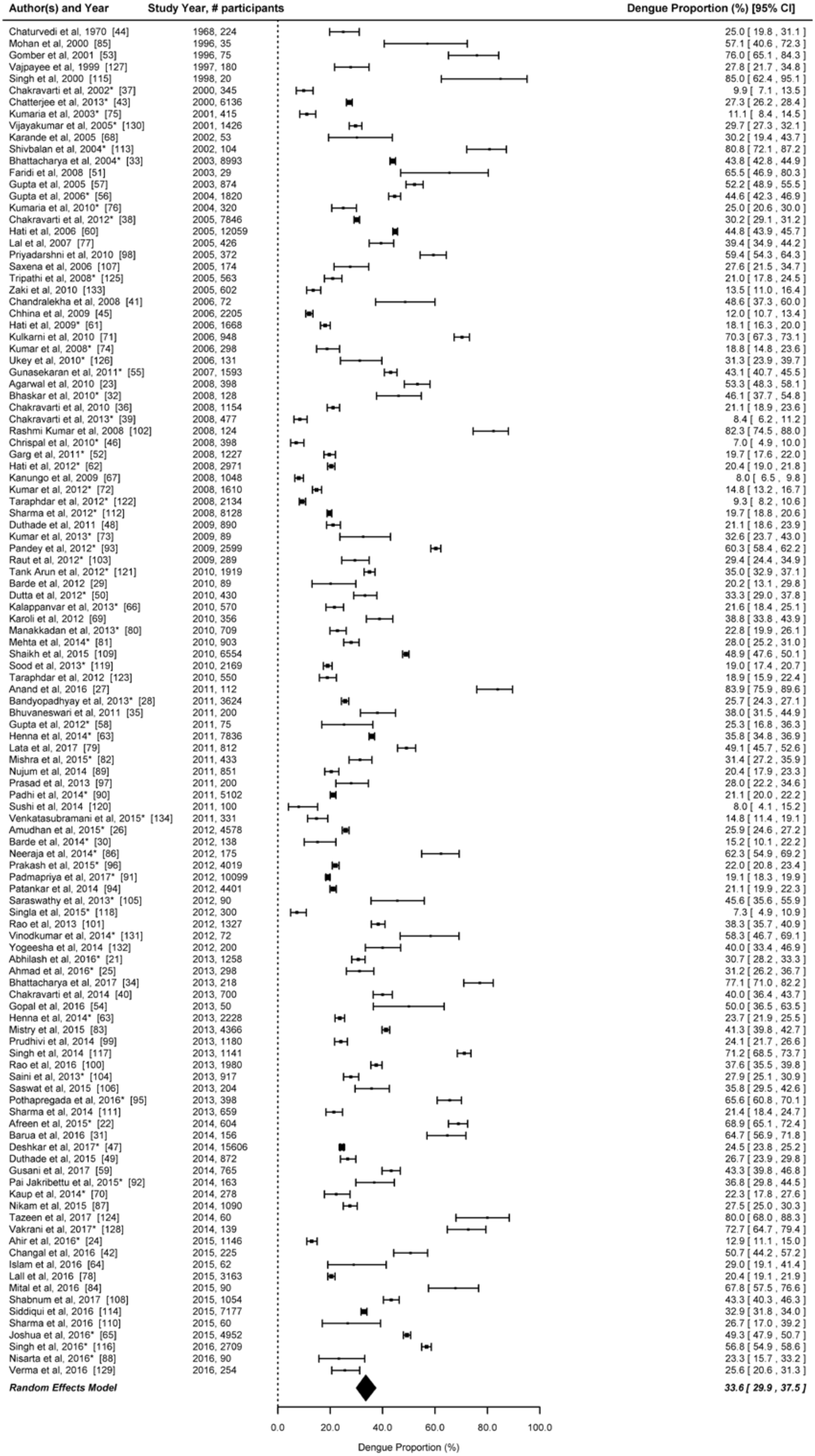

B. Prevalence of lab confirmed dengue in hospital based studies during outbreak or outbreaks

Author(s) and Year	Study Year, # participants	Dengue Proportion (%) [95% CI]
Mathew et al, 1977 [185]	1974, 46	80.4 [66.5 , 89.5]
Rao et al, 1985 [167]	1982, 36	55.6 [39.3 , 70.7]
Mehendale et al, 1991 [186]	1988, 117	56.4 [47.3 , 65.1]
Pushpa et al, 1998* [164]	1989, 58	84.5 [72.8 , 91.7]
Mahadev et al, 1997 [184]	1992, 25	52.0 [33.1 , 70.4]
Baruah et al, 1996 [177]	1994, 14	64.3 [37.6 , 84.3]
Aggarwal et al, 1998 [135]	1996, 39	79.5 [64.0 , 89.4]
Anuradha et al, 1998 [139]	1996, 143	63.6 [55.5 , 71.1]
Dar et al, 1999 [147]	1996, 149	18.1 [12.7 , 25.1]
Kabra et al, 1999 [156]	1996, 58	89.7 [78.8 , 95.3]
Gill et al, 1997 [179]	1996, 9	77.8 [42.1 , 94.4]
Katyal et al, 2000 [182]	1997, 30	26.7 [13.9 , 45.0]
Ratho et al, 2006* [194]	1999, 338	46.7 [41.5 , 52.1]
Rajendran et al, 2006 [192]	2000, 16	81.2 [55.3 , 93.8]
Narayanan et al, 2002 [161]	2001, 89	66.3 [55.9 , 75.3]
Parida et al, 2002 [163]	2001, 312	42.6 [37.3 , 48.2]
Paramasivan et al, 2006 [190]	2002, 57	15.8 [8.4 , 27.6]
Ratho et al, 2005 [193]	2002, 218	34.9 [28.8 , 41.4]
Sarkar et al, 2010 [197]	2002, 781	57.1 [53.6 , 60.5]
Chakravarti et al, 2005 [146]	2003, 1550	37.4 [35.0 , 39.9]
Dar et al, 2006 [148]	2003, 42	19.0 [9.8 , 33.7]
Itha et al, 2005 [154]	2003, 45	80.0 [65.8 , 89.3]
Shah et al, 2004 [169]	2003, 51	76.5 [63.0 , 86.1]
Singh et al, 2005 [170]	2003, 171	70.2 [62.9 , 76.6]
Paramasivan et al, 2006 [188]	2003, 76	19.7 [12.3 , 30.2]
Samuel et al, 2007 [195]	2003, 90	14.4 [8.6 , 23.3]
Hoti et al, 2006* [153]	2004, 92	43.5 [33.7 , 53.7]
Neeraja et al, 2006 [174]	2004, 260	81.2 [75.9 , 85.5]
Banerjee et al, 2007 [141]	2005, 23	8.7 [2.2 , 28.9]
Batra et al, 2007* [142]	2005, 499	5.0 [3.4 , 7.3]
Dutta et al, 2008 [151]	2005, 308	28.2 [23.5 , 33.5]
Paramasivan et al, 2010 [191]	2005, 77	39.0 [28.8 , 50.2]
Bharaj et al, 2008 [143]	2006, 69	69.6 [57.8 , 79.2]
Chahar et al, 2009 [145]	2006, 69	69.6 [57.8 , 79.2]
Gill et al, 2008 [152]	2006, 701	36.5 [33.0 , 40.2]
Rai et al, 2008 [165]	2006, 399	84.7 [80.8 , 87.9]
Vedpathak et al, 2011 [200]	2006, 36	8.3 [2.7 , 22.9]
Dash et al, 2011 [150]	2007, 275	44.0 [38.2 , 49.9]
Neeraja et al, 2013 [162]	2007, 713	37.0 [33.6 , 40.6]
Goel et al, 2009 [180]	2007, 36	13.9 [5.9 , 29.3]
Paramasivan et al, 2010 [189]	2007, 25	52.0 [33.1 , 70.4]
Anoop et al, 2010 [138]	2008, 75	49.3 [38.2 , 60.5]
Sankari et al, 2012* [196]	2008, 58	46.6 [34.2 , 59.3]
Anita et al, 2012* [137]	2009, 107	59.8 [50.3 , 68.7]
Vijayachari et al, 2011 [173]	2009, 101	25.7 [18.2 , 35.1]
Ahmed et al, 2015 [136]	2010, 4370	38.9 [37.5 , 40.4]
Arya et al, 2011 [140]	2010, 1886	45.0 [42.7 , 47.2]
Chaaithanya et al, 2012 [144]	2010, 516	15.5 [12.6 , 18.9]
Mittal et al, 2012 [159]	2010, 219	61.6 [55.0 , 67.8]
Ramachandran et al, 2016 [166]	2010, 1666	55.8 [53.4 , 58.2]
Singh et al, 2012 [172]	2010, 432	12.5 [9.7 , 16.0]
Das et al, 2013* [149]	2011, 185	71.9 [65.0 , 77.9]
Jindal et al, 2014 [155]	2011, 1787	32.8 [30.7 , 35.0]
Mohapatra et al, 2012 [160]	2011, 469	78.3 [74.3 , 81.8]
Saha et al, 2012 [168]	2011, 480	30.6 [26.7 , 34.9]
Khan et al, 2014 [157]	2012, 164	65.2 [57.6 , 72.1]
Barde et al, 2015 [175]	2012, 247	46.6 [40.4 , 52.8]
Biswas et al, 2014 [178]	2012, 100	79.0 [69.9 , 85.9]
Saravanan et al, 2016 [198]	2012, 600	43.3 [39.4 , 47.3]
Barde et al, 2015 [176]	2013, 648	49.5 [45.7 , 53.4]
Khan et al, 2013 [183]	2013, 282	32.3 [27.1 , 37.9]
Muruganandham et al, 2014 [187]	2013, 23	56.5 [36.3 , 74.8]
Shobha et al, 2014 [199]	2013, 68	19.1 [11.4 , 30.2]
Kartick et al, 2017 [181]	2014, 62	43.5 [31.8 , 56.0]
Laul et al, 2016 [158]	2015, 192	59.9 [52.8 , 66.6]
Singh et al, 2016 [171]	2015, 1100	36.4 [33.6 , 39.3]
Random Effects Model		***47.3 [40.9 , 53.8]***

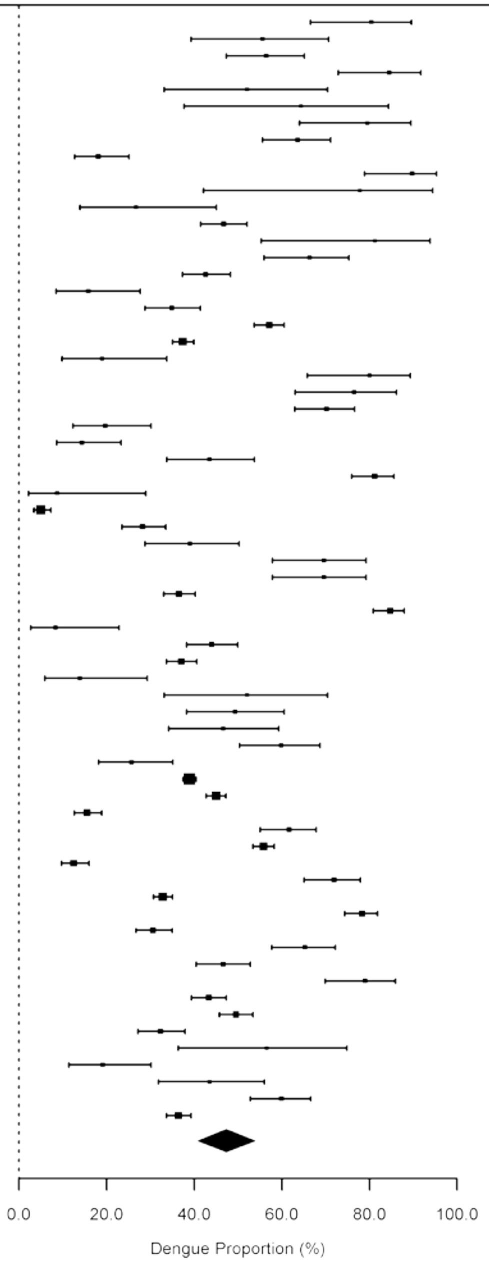

Supplement: S1 Fig — (PDF) [file pntd.0006618.s005.pdf]
